# Fine-tuning of language models for automated structuring of medical exam reports to improve patient screening and analysis

**DOI:** 10.1038/s41598-025-05695-6

**Published:** 2025-07-04

**Authors:** Luis B. Elvas, Rafaela Santos, João C. Ferreira

**Affiliations:** 1 Department of Logistics, Molde, University College, Britvegen 2, 6410 Molde, Norway; 2 ISTAR, Instituto Universitário de Lisboa (ISCTE-IUL), 1649-026 Lisbon, Portugal; 3 Inov Inesc Inovação – Instituto de Novas Tecnologias, 1000-029 Lisbon, Portugal; 4 Breast Cancer Research Program, Champalimaud Foundation, Lisbon, Portugal

**Keywords:** Natural Language processing (NLP), Data mining, Language models, Healthcare, Named entity recognition (NER), Information technology, Scientific data, Data mining, Machine learning

## Abstract

The analysis of medical imaging reports is labour-intensive but crucial for accurate diagnosis and effective patient screening. Often presented as unstructured text, these reports require systematic organisation for efficient interpretation. This study applies Natural Language Processing (NLP) techniques tailored for European Portuguese to automate the analysis of cardiology reports, streamlining patient screening. Using a methodology involving tokenization, part-of-speech tagging and manual annotation, the MediAlbertina PT-PT language model was fine-tuned, achieving 96.13% accuracy in entity recognition. The system enables rapid identification of conditions such as aortic stenosis through an interactive interface, substantially reducing the time and effort required for manual review. It also facilitates patient monitoring and disease quantification, optimising healthcare resource allocation. This research highlights the potential of NLP tools in Portuguese healthcare contexts, demonstrating their applicability to medical report analysis and their broader relevance in improving efficiency and decision-making in diverse clinical environments.

## Introduction

The expansion of Artificial Intelligence (AI) in healthcare has been driven by the increasing complexity and volume of data in this sector^1^. Furthermore, the healthcare sector is facing increasing pressure to enhance the quality of care while contending with time constraints and a growing workload^2^. In this context, AI has become an increasingly valuable solution, with the potential to revolutionise the healthcare system and address its escalating demands^2^.

In hospital settings, large amounts of data are generated, predominantly in textual format, and this volume is expected to continue growing^3^. However, the effectiveness of AI technologies is contingent upon the type of data available. Textual datasets present an additional challenge due to their unstructured nature, which makes it difficult to apply algorithms directly.

To address this challenge, it is essential to implement an Information Extraction (IE) process that transforms unstructured data into a coherent, structured format that represents entities and their relationships^4,5^, thereby producing an organised and coherent representation of entities and their connections^5^. The combination of data mining techniques is of significant importance in the context of pre-processing and structuring medical texts^6^. These techniques facilitate the application of Machine Learning (ML) and Deep Learning (DL) algorithms, enabling accurate predictions, classifications and extractions from structured data^7^.

Moreover, Natural Language Processing (NLP) techniques enable the transformation of unstructured clinical data into structured formats, improving data accessibility and enhancing analysis and interpretation, which provides valuable information for healthcare professionals and researchers^8^. For instance, this method enables the rapid screening of patients with a specific condition, such as aortic stenosis, thereby facilitating studies on the progression of the disease and the screening of patients for future research, which is an example of one of the identified needs.

To fully leverage the vast quantity of data available, it is vital to implement efficient and automated IE techniques. NLP enables computers to understand and extract meaningful insights from textual data^9^. Developing tools to assist in interpreting cardiology reports relies on ML and DL techniques, which are integral to this process^10^. Moreover, the accuracy of these tools is contingent upon the quality of the data; the more structured the data, the higher the accuracy of the tools^11^, and recent studies indicate considerable potential for leveraging these technologies^12^. Technological advancements have led to innovative solutions in healthcare, assisting in diagnosis, screening and individual patient assessment^13^. Analysing medical reports can expedite diagnoses and enhance the efficacy of treatments^14^.

In addition, research into fine-tuning applied to text extraction allows the models to go beyond basic keyword extraction and entity recognition to understand the deeper context of specific domains, making text extraction more effective and accurate in specialised applications^15^. However, models based on unstructured text data are challenged by the lack of annotated data, which is critical for effective algorithm training^14,16^. By doing so on specific medical data, such as cardiology, the models can learn to recognise and prioritise the most relevant terms and patterns for that domain, improving the accuracy and reliability of the analysis^16^.

As mentioned, this research was guided by a need identified by Medical Doctors (MDs) at Hospital de Santa Maria. Faced with the challenge of conducting a manual and time-consuming survey of diseases from clinical reports, the MDs highlighted the need for an automated and efficient approach to screening.

In this manner, the processing of medical reports derived from the Portuguese hospital was undertook. To do so, language models capable of extracting and structuring entities were utilised and fine-tuned, specifically ‘disease’ and ‘diagnosis’ related. These entities were subsequently incorporated into a data frame designed for integration with an interactive Power BI report. This report enables MDs and hospital managers to track disease and diagnosis metrics, addressing the challenge of analysing unstructured information on a large scale.

**Fig. 1:**
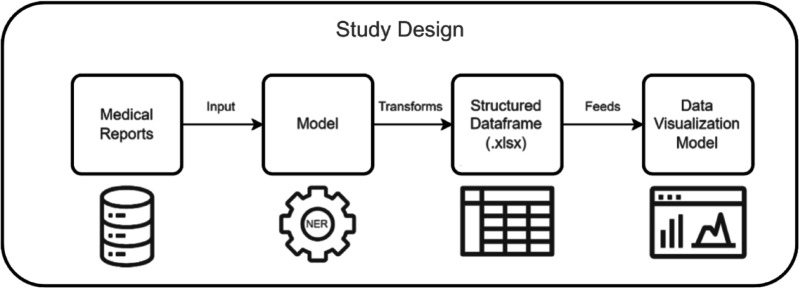
Implemented approach for the identified problem.

Figure 1 provides an overview of the study design, illustrating the sequential flow from data collection to the final analytical result. The design commences with the initial phase of receiving medical reports, which are then subjected to NLP techniques, particularly Named Entity Recognition (NER), to identify and extract pertinent medical entities, such as diagnoses and diseases. The extracted data is methodically organised into a structured data frame, which is used to feed a visualisation tool, thereby enhancing the capacity to explore and interpret the results interactively.

Currently, the average time required to analyse each medical report is approximately seven minutes. For the 12,651 reports that can be accessed, this would amount to 1,475.95 h or 61.5 days, which equates to 210.85 working days. If a physician were to dedicate three hours daily to this task, it would take 492 days (excluding holidays and vacations). The simultaneous work of three physicians would be needed to complete this task in approximately six months.

The primary objective of this investigation is to develop advanced methodologies to extract meaningful knowledge from medical textual reports through Natural Language Processing (NLP) and Machine Learning (ML) techniques^17,18^.

Traditional manual analysis methods are inherently time-consuming and prone to human error. In contrast, automation and advanced computational models offer a more agile and accurate approach to processing medical information. The proposed methodologies will facilitate improvements in patient selection, clinical studies, decision-making and research by enabling more efficient and precise screening of medical reports.

This paper aims to analyse cardiology medical reports from Hospital de Santa Maria in Lisbon, Portugal, to identify diseases and diagnoses based on the data contained in the reports. This analysis aims to achieve two primary goals (G):

- G1. Facilitate patient screening by efficiently identifying relevant medical conditions;
- G2. Establishing a relationship between diagnoses and diseases based on the available data.

The research employs data mining techniques to structure unstructured medical reports, identify key entities - diseases and diagnoses - and extract valuable information from the data. In this context, diseases refer to specific medical conditions, such as heart failure or aortic stenosis, that are documented within the reports as underlying health issues. Diagnoses, on the other hand, represent the clinical conclusions or assessments made by healthcare professionals based on the patient’s presentation and test results, often indicating the presence or status of a disease.

To achieve these objectives, the information derived from the medical reports was structured, and pre-trained language models were fine-tuned using this specific dataset. This fine-tuning process adapted the models to the particularities of the dataset under investigation, enhancing their ability to extract relevant medical information. This will enable to answer the following Research Question (RQ):

- To what extent can fine-tuned language models, optimised through hyperparameter tuning and trained on a medical lexicon, effectively perform NER to identify diagnoses and diseases in cardiology-related medical imaging reports in non-English languages, with Portuguese as a test case, for clinical applications such as patient screening?

This research question aims to assess the feasibility of applying these models in clinical decision support and patient screening, with the goal of extending their applicability to other non-English medical contexts.

The methodology employed to address the data mining challenges follows the Cross-Industry Standard Process for Data Mining (CRISP-DM)^19^, comprising six iterative phases. The methodology begins with Business Understanding (Sect. 3.1.), identifying Pre-Trained Models (PTMs) with promising performance for disease prediction. The Data Understanding phase involves describing and assessing data quality, while the Data Preparation phase (Sect. 3.2.) focuses on critical tasks such as tokenization, manual annotation of entities such as diseases and diagnoses and data cleansing. The Evaluation phase (Sect. 4.2.) reviews model performance, potentially involving hyperparameter tuning, and the final Deployment phase includes creating a visualisation dashboard to explore results from applying the PTMs.

## Related work

Data mining has emerged as a crucial tool for extracting pertinent information from voluminous medical data, just as NER techniques play a pivotal role in automatically identifying and categorising relevant information in clinical texts, including diagnoses, medications and symptoms, thereby facilitating a more detailed and structured analysis of medical reports^20^. According to Durango et al.^20^, fine-tuning enables the customisation of pre-trained AI models for specific applications, thereby enhancing their accuracy and adaptability to the nuances of medical data.

The topics addressed on the papers selected have been extracted for analysis, including data mining, big data, NLP, text extraction and Pre-Trained Models (PTMs).

This approach allows for a clear and structured organisation of the articles, facilitating an understanding of key research directions. Table 1 presents an analysis of recurring topics in literature reviews, highlighting dominant themes and their prevalence. This helps to identify major research trends and gaps in the intersection of health and information technology, with a particular focus on emerging methodologies such as pre-trained language models and AI applications in medical data.

**Table 1:** Topics found in literature reviews.

Topic	Reference	Number of documents
PTMs	^61–74,76^	15
NLP	^50,52–57^	7
Big data and AI applications	^38,39,46–49^	6
Data mining	^34–37^	4
Text extraction	^42–44^	3
Health research methods	^40,41^	2

**Fig. 2:**
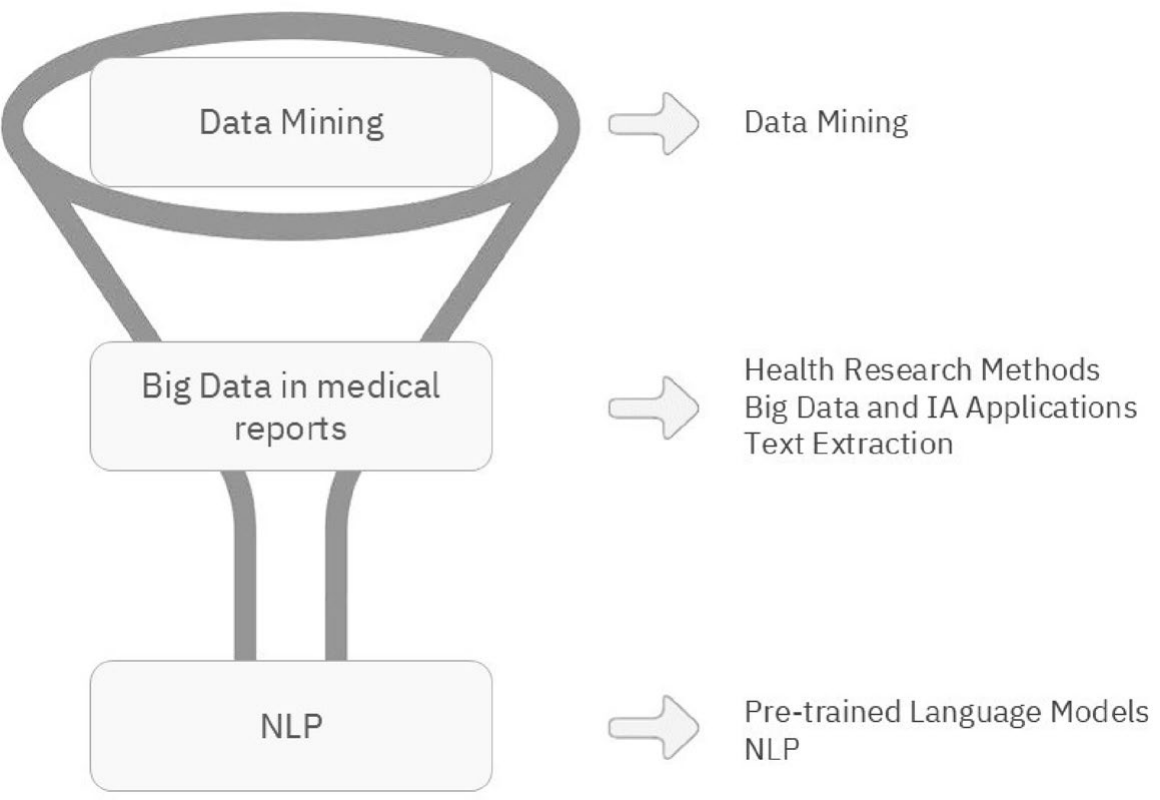
Organisation of literature review topics.

As shown in Table 1, the most frequently addressed topics involve advancements in NLP and AI applications, particularly in medical data processing. Given the prominence of these themes, the literature review was structured to reflect the hierarchy of research focus. Accordingly, the main themes were categorised into data mining, big data applications in medical reports and NLP, as illustrated in Fig. 2.

This structured approach was chosen to clarify the intricate relationships between these areas and to provide a comprehensive understanding of their interconnectedness. To achieve this, individual topics were consolidated into broader themes according to their relevance and the connections between them. These topics were systematically categorised according to the following outlines:

- Data mining : This topic involves extracting useful patterns, knowledge and ideas from large data sets. It is important in the context of this research, particularly in analysing textual data from reports. This process can also be referred to as text mining;
- Big data in medical reports (this theme includes the topics big data and AI applications, text extraction, health research methods and IoT in healthcare): This topic presents an investigation into the transformative impact of big data analytics in the field of medical reporting, with a particular emphasis on the advancement of AI applications within the context of medicine;
- NLP (this theme includes the topics: PTMs and NLP):
  - Methods and applications: This focuses on the practical use of NLP techniques to process medical texts, such as extracting information from clinical narratives and reports. It emphasises methodologies that manage large volumes of unstructured text data, essential for improving healthcare delivery;
  - Techniques and models: This explores specific NLP models and techniques, particularly PTMs in healthcare. It highlights recent advancements in NLP and how these sophisticated models enhance the analysis and interpretation of medical documents.

### Data mining

Data mining in healthcare is crucial for extracting valuable patterns and knowledge from large volumes of data, according to^21^, the medical field is at the forefront of technological innovation, seeking new strategies to diagnose, treat and prevent diseases. According to^22^, this science involves analysing data, in this case textual data, using mathematical models to extract knowledge and gain insights.

Predictive modelling, a key ML application in healthcare, predicts diagnoses and patient outcomes from clinical data, and according to^23^, these models assist in diagnosing diseases, assessing severity and understanding complication risks, leading to more effective treatment.

AI-based text mining tools process data, exploring characteristics and correlations, according to the study^7^, an EdIE-R system has been developed that labels medical reports with specific observations from electronic health records^24^, such as small vessel disease or stroke, through techniques such as NER which is used to identify words or phrases that are relevant ‘entities’ for the text mining task, and relation extraction is used to identify and link relevant entities in medical texts.

The author of^25^ argues that the careful design and systematic application of advanced AI methods, such as NLP and ML, have the potential to bridge knowledge gaps, and a concrete example of this is the study mentioned, where the use of NLP on a database of 844,683 medical records in the United States resulted in a doubling of hypoglycaemia diagnoses compared to traditional methods, as well as an over twenty-fold increase in diagnoses of non-serious events.

### Big data in medical reports

According to^26^ there is currently an exponential growth in data volume, encompassing both textual and image formats, which has sparked interest in leveraging big data methodologies to analyse and extract insights from vast and complex datasets spanning the entire spectrum of healthcare. These methodologies promise to streamline labour-intensive tasks traditionally performed by radiologists^27^.

Addressing the considerable costs inherent in radiology involves the breakdown of expenses^28^, which are subdivided into technical fees, covering the costs of acquiring imaging apparatus and devices, and professional fees attributed to radiologists for the interpretation of medical images. Consequently, the advent of text extraction^29^, emerges as a crucial process, transforming unstructured textual data into structured formats suitable for integration into databases, data warehouses or business intelligence platforms^30^.

The transformation of unstructured clinical narratives into structured data begins with information extraction, facilitated by methodologies such as NLP^9,31^, which enable the automatic identification and extraction of pertinent information, facilitating a seamless transition into structured datasets.

In the work^32^, the radiology department can identify patient symptoms by gathering relevant data from medical applications and storing it in the electronic health record system using IoT technology, and by integrating it with AI, patient tracking can be done promptly, which in turn facilitates informed decision-making.

However, the inherent challenge in processing free-text formats, characterised by linguistic ambiguity, necessitates innovative solutions. The author of^33^ developed ML approaches and achieved an average cross-validation efficiency of 85% in information extraction.

According to^34^, in the field of computer-aided detection and diagnosis, significant strides have been made in mitigating errors in the interpretation of medical images. Leveraging both image data and labels predefined by healthcare professionals, structured reports, NLP and ontologies were utilised to enhance the analysis and generation of imaging procedures, thereby promoting a more informed clinical practice.

According to reference^35^, this application has already been integrated into a research image viewer and picture archiving and communication system platform, by incorporating algorithms, the application streamlines image export tasks, facilitates the generation of segmentations and annotations and enables clinical production tests. This prevents wastage of time and resources in data conversion and transfer and allows the focus to be solely on the performance of the algorithm, which serves as an aid.

The study^36^ promotes resource allocation based on data analysis, heralding it as a paradigmatic shift through the development of predictive models, reduction of waiting times and optimisation of patient outcomes via data mining, ML and predictive analysis.

This chapter underscores the transformative potential of the amalgamation of AI, NLP and big data in revolutionising clinical practice, fostering enhanced diagnostic efficiency and facilitating personalised patient care.

### NLP – methods and applications

According to the authors^37^, NLP methods can help computer systems understand information just like humans, thereby optimising knowledge and scaling clinical response. This application has been instrumental in transforming the analysis of.

medical reports.

Key NLP technologies for these tasks include pattern matching and linguistic analysis. The author^38^ explored text analysis and knowledge extraction using Text Analysis and Knowledge Extraction (cTAKES)^39^, an NLP system designed for clinical texts. It identifies concepts and assigns a “polarity” to indicate whether a finding is present or absent.

According to this article^38^, its result by simple pattern matching had an accuracy of 97% in detecting recommendations for additional imaging for incidental findings in 1635 emergency department radiology reports and an accuracy of 99.6% in detecting recommendations in 1059 consecutive reports across all imaging modalities in an academic hospital radiology department.

For the use of deep neural networks to classify radiology text reports, author’s^40^ recommends that the text must be firstly converted into a numerical format. This procedure is called language embedding or modelling, and by adapting a language model to the radiology “language” can significantly improve the results of text classification models.

In the work^41^, it is presented an experiment using these and other NLP applications, such as tokenization, stemming and lemmatisation of words (simplifying terms related to a common root by assigning them a similar meaning or value, grouping semantically related words for more efficient analysis), stop words (removing words without semantic relevance, reducing noise), vectorisation (converting free-text information into numerical values, using models such as “Bag of Words” and “Word2Vec”), and Bidirectional Encoder Representations from Transformers (BERT). The study^42^ demonstrates the effectiveness of calculating cardiologists’ compliance rates in the use of standardised reports, whereby the average compliance rate obtained through automated NLP auditing reached an average compliance rate of 92.0%.

Regarding NLP libraries, according to^43^, the Natural Language Toolkit (NLTK)^44^ encompasses all components of a typical NLP pipeline. SpaCy^45^, while less comprehensive than NLTK, provides all major NLP components while also emphasising speed and production. The integration of models, including DL models, into the SpaCy framework is straightforward and has become popular for both production and research projects. Additionally, projects such as scispaCy^46^ are built upon SpaCy for analysing scientific texts.

### NLP – techniques and models

This section explores key advancements in NLP models, focusing on both general techniques and domain-specific applications, including adaptations for the Portuguese clinical context.

#### General NLP models and techniques

In the work^47^, BERT is a pre-trained, bidirectional language representation model that operates without relying on a specific input text sequence, finding various applications. In the radiology domain, RadBERT stands out, trained on millions of radiology reports.

Authors of^43^ use BERT as an experience/case study by randomly selecting approximately 15% of words to replace with a “masked” token, then attempting to predict the masked tokens. The article^40^ emphasises the use of Word2Vec to convert words into numerical vectors, where words with similar contexts have similar vectors, differing from the CBOW model, which observes words near the target word to predict its context, and the skip-gram model, which predicts the context of the target word from itself. This approach highlights^48^ the diverse strategies employed by these neural network architectures in capturing semantic relationships within textual data.

In^49^, RadBERT-CL is explored, an algorithm developed for multi-label classification, resulting from fine-tuning the BlueBERT model, another BERT variant^50^, on the MIMIC-CXR dataset of 377,110 chest X-ray reports. Another tool, BI-RADS BERT, was designed to segment a report into standard sections (e.g., “Findings,” “Impression”) and extract information from specific fields, developed by fine-tuning BERT on 155,000 breast imaging reports.

The article^51^ proposes an approach based on transformers, using language models such as BERT and RoBERTa to perform named entity extraction, which developed an annotation scheme and a specific corpus for breast cancer in Spanish, achieving F scores of over 90%.

In the work^54^, authors utilised the BERT model and clustering algorithms to organise similar documents into distinct topics, incorporating both RadLex and SentiWordNet. The combination of cTAKES, RadLex and the general-purpose dictionary resulted in an F-measure of 30.9% (precision of 73.3% and recall of 19.6%), while the use of the enhanced compound term dictionary achieved an F-measure of 63.1% (precision of 82.8% and recall of 51%).

The article^55^ delved into Bidirectional Encoder Representations from Transformers for Biomedical Text Mining (BioBERT), a domain-specific language representation model pretrained on extensive biomedical corpora. With a nearly identical architecture across tasks, BioBERT outperformed BERT in three representative biomedical text mining tasks: biomedical NER (0.62% improvement in F1-score), biomedical relation extraction (2.80% improvement in F1-score) and biomedical question answering (12.24% improvement).

The authors^56^ trained the BERTimbau model on a Brazilian Portuguese dataset, which outperformed Multilingual BERT.

In the work^57^, the authors explored the BrainNERD model for accurately extracting acute brain injury terms and their properties from head CT reports, which included 1152 patients in the training set-,Ten-fold cross-validation performance was 93–99%, and NER test performance metrics were 98.82% for precision, 98.81% for recall and 98.81% for F1-score.

In^58^, the extraction of lesion findings and medical issues from radiology reports was developed. For argument entity extraction, two state-of-the-art neural architectures were evaluated, using BiLSTM-CRF and BERT. The pre-trained BERTrad model on three million radiology reports achieved an average F1 score of 85.5% at the token level. Next, clinical finding events were extracted by predicting argument roles for the extracted entities. Overall, the average F1-scores for event extraction were 92.9% for triggers, 75.0% for extension-only arguments and 84.8% for extension arguments with values.

In the work^59^, RadText, a high-performance, open-source Python radiology text analysis system, offers a text analysis pipeline, de-identification, section segmentation, sentence splitting, word tokenization, NER, syntactic analysis and negation detection, demonstrating highly accurate classification performance with an average precision of 0.91, average recall of 0.94 and average F-1 score of 0.92.

The author^60^ delved into individual masked deep language models, achieving an overall macro F1-score performance of 77%, representing an effective strategy for addressing NER in corpora within the healthcare and life sciences domains.

The article^40^ developed language modelling using the Universal Language Model Fine-Tuning (ULM-FiT) technique^61^, which fine-tunes a PTM, a different technique from the use of BERT. Classifier training was conducted progressively, starting from the last layers and progressing to the upper layers. Performance analysis was carried out on the test set, using metrics such as precision, recall and F1-score. A confusion matrix was plotted to visualise false positives and false negatives, while “top loss” analysis highlighted data points with the highest discrepancy between predictions and labels. Ultimately, the results and applications showcased the utilisation of the fastai library^62^ for processing radiology reports, training models to classify reports as either normal or abnormal.

The authors of^63^ declare that both techniques are valid, and BERT utilises transformers and stands out for its “transformer” architecture and its effectiveness in capturing bidirectional context, whereas ULM-FiT employs long short-term memory networks and is recognised for its effective fine-tuning approach across a variety of tasks with limited training data.

#### NLP for clinical text in Portuguese

Despite significant advancements in clinical NLP, most models are trained in English, with limited domain-specific adaptations for Portuguese. Addressing this gap, researchers have developed Portuguese-specific clinical language models, including Albertina PT^52^ and MediAlbertina^53^.

- Albertina PT: A transformer-based model trained on Portuguese-language datasets, using DeBERTa as its foundation;
- MediAlbertina: A clinical adaptation of Albertina PT, specifically trained on Portuguese healthcare texts, demonstrating superior entity recognition performance in cardiology reports.

These models represent a significant advancement in Portuguese-language clinical NLP. The MediAlbertina model, specifically trained on European Portuguese, has demonstrated superior performance compared to Brazilian Portuguese models in entity recognition tasks, highlighting the importance of fine-tuning language models to Portugal’s specific clinical context^53^. However, further research is needed to evaluate their effectiveness in real-world clinical settings and to assess their tangible benefits in practice. A critical challenge remains the limited availability of annotated Portuguese clinical datasets, which must be expanded to enhance model performance across various medical specialties.

### Discussion

A review of the key points in each section reveals that Sect. 2.1 on data extraction is of paramount importance in the field of healthcare. This section elucidates the indispensable role of data extraction in the discovery of valuable patterns and knowledge from extensive data sets. Predictive modelling, a pivotal application of ML enables the prediction of diagnoses and patient outcomes, thereby facilitating the formulation of more efficacious treatment plans. AI-based text extraction tools, such as NER recognition and relation extraction, demonstrate considerable potential in the efficient processing and analysis of medical texts.

The following section, 2.2, examines the application of big data in healthcare, with a particular focus on medical reports. As the volume of data continues to grow exponentially, it is becoming increasingly important to understand how big data methodologies can be continually reviewed and improved to keep pace with technological advances. However, the integration of unstructured clinical narratives into structured data remains a significant challenge that requires further investigation.

Subsequently, in Sect. 2.3, the NLP techniques described therein represent a viable approach to text processing in healthcare. The main techniques for the project include text pre-processing (tokenization and removal of illegal characters), Part-Of-Speech (POS) analysis and manual annotation according to defined tags. The use of SpaCy can facilitate and accelerate the development of DL models.

In the final section, 2.4, the various models are presented, along with their applications and the results obtained. While BERT-based models such as RadBERT and BioBERT have demonstrated high accuracy in English clinical texts, their direct application to Portuguese data is limited.

Deep learning models such as BERT and BioBERT improve diagnostic precision. The introduction of MediAlbertina demonstrates the potential for language-specific fine-tuning, but additional datasets and validation studies are needed to bridge the gap with English-trained models. The extraction of data and the utilisation of NLP tools, such as NLTK, are fundamental for the analysis of textual data.

In general, the results are positive, demonstrating high accuracy and efficiency. Notable advancements have been observed in models such as Albertina and its derivatives including MediAlbertina and BERT (e.g., BERTimbau and RadText).

The analysis demonstrates that models such as BERT and its variants hold considerable potential for medical text analysis, exhibiting high levels of accuracy and efficiency. However, they face significant challenges when applied to different languages and clinical contexts, primarily due to the need for linguistic adaptation. This represents a critical gap, as a substantial proportion of these models are trained on specific corpora that fail to encompass global linguistic diversity, thus limiting their universal applicability.

Another challenge is the inherent complexity of medical texts, which are often ambiguous and difficult to interpret. While advanced NLP methods, in conjunction with big data, offer promising solutions to these difficulties, it is essential to maintain a continuous effort to adapt these tools to different clinical scenarios effectively.

The integration of AI, NLP and big data can revolutionise clinical practice. Nevertheless, ongoing advancements are necessary to guarantee their efficacy across diverse contexts and realities.

## Data exploration and Preparation

**Fig. 3:**
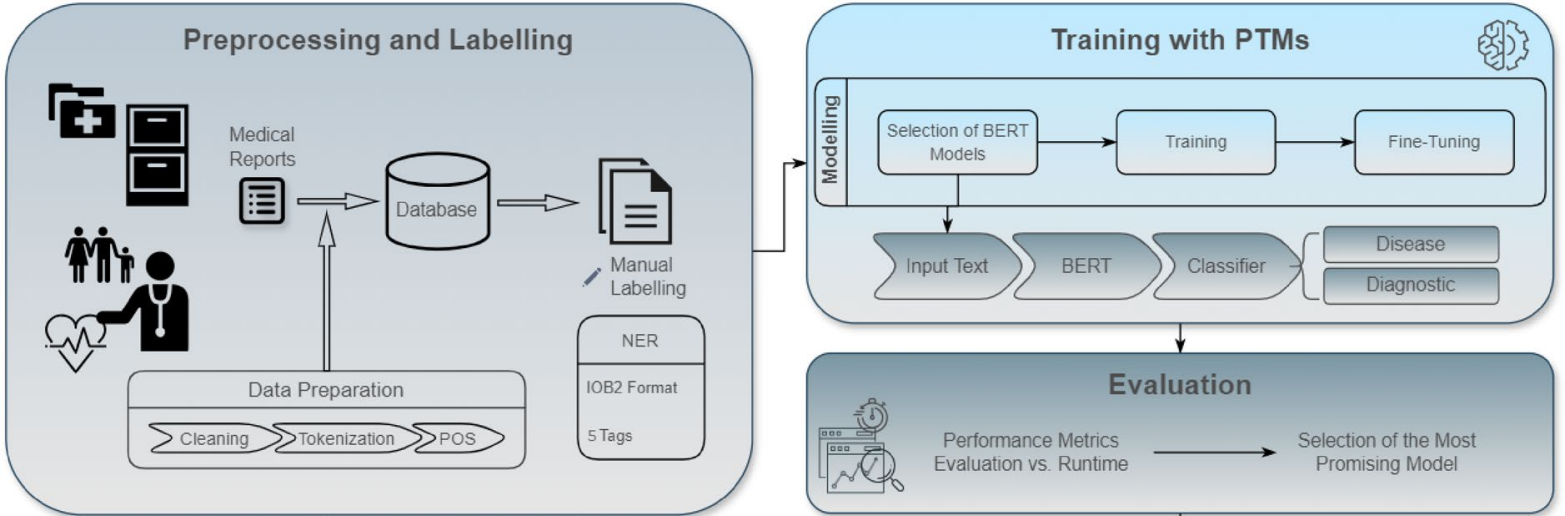
Identified initial steps to achieve the identified goals in this research.

The research process was conducted in accordance with the methods delineated in the literature review, as depicted in Fig. 3. The image is divided into three principal phases, which are detailed in Sects. 3, 4 and 5, respectively.

In the initial phase, medical reports are collated and stored in a database. This stage entails preparing the data through cleaning, tokenization and POS tagging. Subsequently, manual annotation is applied to identify the entities pertinent to the study, such as diseases and diagnoses, using the IOB2 format with five types of tags.

The second phase is dedicated to the using of PTMs. In this stage, BERT models are selected, and the model is then fine-tuned to ensure accurate classification and identification of the entities extracted from the medical reports.

In the final phase, the performance of the trained models is subjected to rigorous evaluation based on performance and runtime metrics. This process aims to identify the most promising model that offers an optimal balance between efficiency and accuracy.

### Business and data Understanding

This research aims to develop a classification system for medical reports, facilitating the screening process. It is therefore essential to organise the information contained in cardiology reports in order to identify terms for the disease category.

The dataset under examination was sourced from Hospital de Santa Maria, Portugal’s largest public hospital, and provided by the cardiology department. The FCT project DSAIPA/AI/0122/2020 AIMHealth-Mobile Applications Based on Artificial Intelligence provided the actual anonymised data used in this study, which came from the same hospital^64^. All methods were approved by the Faculty of Medicine of Lisbon’s Ethical Committee (February 17th, 2023, reference number 132/22), one of the project partners. All methods were carried out in accordance with relevant guidelines and regulations.

In this study, a total of 12,651 examination reports were collected from November 27th, 2020, until January 26th, 2023, amounting to 38.12 megabytes of data. The data was stored in individual sets, with each medical report represented by a JSON file. These were organised into 12,651 JSON files, resulting in 12,651 medical reports.

The dataset described in Table 2 was composed of a single table, “PACS_Reports,” which included two categories of data: “Patient” and “Report”. The first sub-table, “Patient”, contained information about the patient under analysis. The second sub-table, “Report”, contained details of the medical imaging examination carried out on the patient. Following the coding of the “Observation” and “Report” columns in RTF^65^, the data was converted into text using the “latin-1” coding parameter, due to its sensitive nature.

**Table 2:** Dataset description.

Table	Variable name	Variable description
PACS_Reports	Filename	Report name
PACS_Reports	Extraction_Timestamp	Timestamp at which the data was extracted from the medical records system
PACS_Reports.Patient	ID	Unique identifier assigned to each patient
PACS_Reports.Patient	Birthdate	Patient birthdate
PACS_Reports.Report	AccessionNumber	Internal number of the medical examination carried out
PACS_Reports.Report	Exam_Type	Type of cardiology exam
PACS_Reports.Report	Observation	Patient observations
PACS_Reports.Report	Report	Description of the medical report
PACS_Reports.Report	Validation_Timestamp	Date of medical examination

At this preliminary stage, it was essential to evaluate the quality of the data. This entailed verifying the completeness and accuracy of the data, identifying any discrepancies in formatting and determining whether any values were absent. The first step was to perform a thorough analysis of the data, checking for null values and duplicates, as shown in Table 3.

**Table 3:** Data quality.

Variable name	Variable type	Nulls	Duplicates
Filename	Object	0	0
Extraction_Timestamp	Object	0	–
ID	Int64	0	11,110
Birthdate	Object	0	–
AccessionNumber	Int64	0	5349
Exam_Type	Object	1	–
Observation	Object	1655	–
Report	Object	7	–
Validation_Timestamp	Object	511	–

It was interesting to determine the time frame of the medical reports, as indicated by the “Validation_Timestamp” in Table 3, which reflects the dates of the medical examinations. The earliest report was from November 27th, 2020, at 18:41:00, and the latest was from January 26th, 2023, at 12:40:00. This timestamp provides a clear temporal context for the data analysed in this study.

After completing this phase, the data was prepared for the next step, which involves cleaning and processing.

### Data Preparation

In accordance with the CRISP-DM data lifecycle, the objective of this phase was to conduct an analysis and cleansing of the reports, subsequent to the tokenization and annotation process, in order to prepare them for input into the language model. Without this phase, the NER model would not be able to function effectively, therefore, it is a prerequisite for ensuring data compliance.

Considering the variables under investigation, it was determined that part of them, such as “Filename,” “Extraction_Timestamp,” “ID,” and “AccessionNumber” would not bring meaningful contextual information to the study, as they are mapping variables for both the report and the patient.

Following an assessment of the data quality, the most pertinent information for identifying the entities “disease” and “diagnosis” was selected, primarily within the “Observation” and “Report” variables. Additionally, other columns, such as “Birthdate,” “Exam_Type,” and “Validation_Timestamp,” were deemed crucial for contextualising the study, particularly for the screening phase. As part of the data preparation process, the content of the “Observation” and “Report” variables was analysed, and it was found that the information they contained was complementary. As there is no evidence that information about the disease or diagnosis is confined to a single variable, both variables describe a clinical analysis in free-text format.

The variables were therefore combined into a single “full_report,” which brought together all the relevant information from both sources, ensuring that the data was comprehensive and unified for analysis.

Following this procedure, the null values in the data set were then processed. As a result of the creation of the “full_report” variable, seven null values were inherited by the seven null values common to both original variables, and thus excluded to ensure the reliability of the dataset.

Concerning the “Exam_Type” variable, the null value presented could not be reliably filled in, which led to the exclusion of this report. Furthermore, it was determined that the null values in the “Validation_Timestamp” variable had minimal impact on the overall results of the study, which made possible to retain the 511 reports and considered null values for this variable.

No additional formatting modifications were necessary for the existing variables, as their data types were already consistent with the study’s requirements. The newly created “full_report” variable was correctly classified as a string.

An essential aspect of data preparation was the annotation process, which constituted a crucial phase for training the pre-trained models used in the study. The annotation process entailed meticulous manual annotation of specific entities in the text, such as diseases and diagnoses. This annotation provided a solid basis for the models to be trained from reliable reference points.

Subsequently, the data was prepared for the next stage, tokenization, following the completion of the preceding processes of sealing, cleaning and ensuring that the variables align with the research objectives.

### Tokenization

The tokenization process was applied to the concatenated “full_report” variable. This step is fundamental for dividing the text into discrete units, or tokens, for subsequent analysis and processing in PTMs.

The next task of tokenization was undertaken, employing the NLP pipeline in the Portuguese language of spaCy, namely ‘pt_core_news_lg’^66^. This choice is justified by the recognised efficiency of spaCy in processing large-scale text, coupled with its various functionalities, such as tokenization, morphological analysis, syntactic analysis and NER^67^, all of which are essential for the execution of this study.

Particularly noteworthy is that the ‘pt_core_news_lg’ model was selected due to its extensive pre-training with a large amount of Portuguese language data, which provides the model with a prior understanding of the language’s grammatical structure and vocabulary. This tends to result in superior performance in text analysis tasks compared to generic models^68^.

Illegal characters were then removed using the ‘remove_ilegal_chars’ function while employing the regular expression ‘[\x00-\x08\x0B-\x0C\x0E-\x1F\x7F]’ and replaced with blank spaces to ensure data consistency and integrity.

An iterative process was carried out for each line of the data frame, i.e., for each medical report. Each report was transformed into an Excel file, where the tokens extracted from the original “full_report” column were recorded. SpaCy’s ‘nlp’ module processed these to split the text into tokens, resulting in a file with one token per line.

After this step, a column was added to each file with each token’s respective grammatical categories (“POS”).

Therefore, each Excel file had two columns: the first column “Token” contained all the valid tokens found in the report, while the second column “POS” recorded the corresponding grammatical categories.

This organisation enabled the creation of individual Excel files for each report, simplifying the identification and analysis of the data per report. The Excel files were named according to the standard ‘Filename.xlsx’, providing a simple and straightforward way of associating the data with each specific report.

After tokenizing the 305 files, exploratory analysis was conducted to understand the file dimensions, as shown in Table 4.

**Table 4:** Exploratory analysis of tokens by excel file.

File count	Min tokens	Max tokens	Avg tokens	Mode tokens
305	1	1882	253 472	35

### Annotation

The annotation process was one of the most time-consuming and critical phases of this study, requiring approximately ninety hours of continuous and meticulous work. This phase entailed the manual identification of relevant entities in the medical reports, a fundamental step for applying the PTMs, similar to the studies analysed in the literature review.

In order to facilitate a more comprehensive grasp of the subject matter, the following clarifications are presented:

- Disease: Used to describe a medical condition that impairs normal bodily functions (such as heart failure and pericarditis);
- Diagnostic: Used to describe the conclusion or result derived from medical examinations, determining the presence or nature of the disease (such as narrowing of the aortic valve and aorta with nodules).

In this phase, each token in the Excel files generated in the tokenization phase was manually annotated with specific labels. This manual annotation was essential to ensure the accuracy and quality of the data that would be used in the NER models. This work was conducted with a continuous investigation of the terms described in the reports, requiring a detailed and careful understanding of the medical terminology employed.

Accordingly, a “LABEL” column was incorporated into each Excel file, wherein each corresponding token was designated a label. The labels were defined as follows: “D” for diseases and “R” for diagnoses and medical results, as detailed in Table 5.

**Table 5:** Tags of NER using IOB2 format.

Tags	Description
B-D	The beginning of a disease
I-D	Part of a disease
B-R	The beginning of a diagnostic/ medical result
I-R	Part of of a diagnostic/ medical result
O	Not named entity

The annotation scheme helps to represent the position of the token concerning what it represents within the entities^20^. The system implemented was IOB2, similar to IOB (Inside, Outside, Beginning), but a B tag is assigned to each token that exists at the start of the block, instead of being dated at the start of the fragment immediately following another fragment of the same named entity^69^. This decision was based on the slight difference and the already attained results with this structure.

In this context, the IOB2 scheme is a convention widely used in NLP, especially in tasks such as NER, where “B-” (Beginning) indicates that the token is the beginning of the entity, “I-” (Inside), the token is inside the entity that has already begun, and “O” (Outside) indicates that the token does not belong to an entity^70^.

Since this study used subword tokenizers, the annotation process had to be adjusted. Subwords split words into smaller units, so the labels were designed accordingly: the first subword-token of an entity received a “B-” label, and subsequent subwords got an “I-” label. This maintained accurate representation of the entities despite the token splits.

During the annotation process, several issues were identified that could potentially impact the results. One significant challenge was the presence of spelling errors in the medical imaging reports. For instance, the word “chnoque hemorragica” was incorrectly used instead of “choque hemorragico.” After discussing this, it was decided by the authors to treat these errors as spelling mistakes, and the correct term, “choque,” was labeled as “B-R”.

Additionally, it was noted that adjectives associated with the labels, such as in the phrase “densificação ligeira,” (“light densification”) were indeed annotated. In this example, “ligeira” (“light”) was labelled because it provides a significant qualifier to “densificação.” As such, both terms were annotated together as “B-R” and “I-R” respectively, ensuring that the full context of the medical condition was captured accurately.

**Fig. 4:**
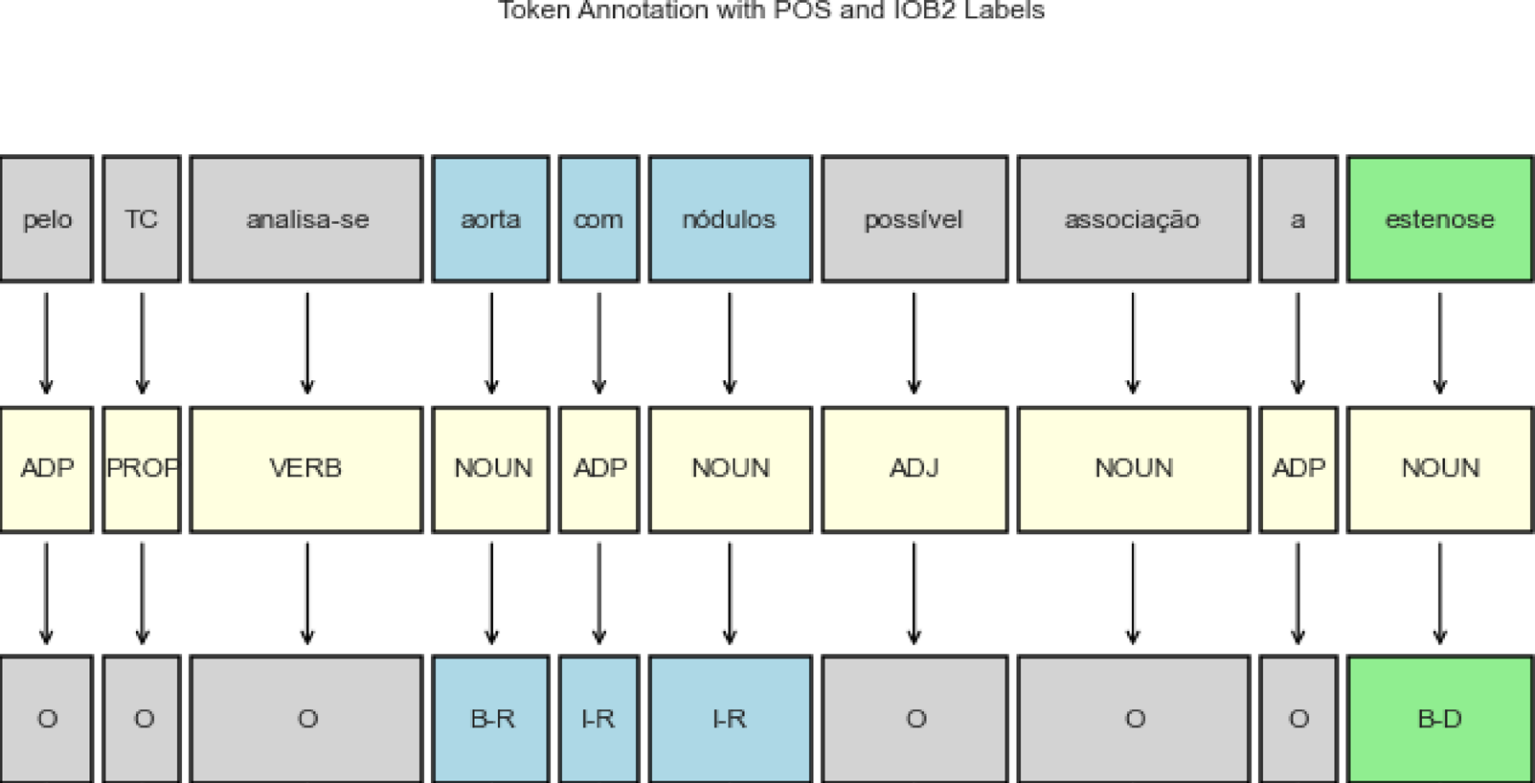
Example of IOB2 annotation at the word-token level.

To illustrate, Fig. 4 depicts the annotation of the phrase “pelo TC analisa-se aorta com nódulos, possível associação a estenose” according to its POS and the corresponding label in the IOB2 schema.

**Fig. 5:**
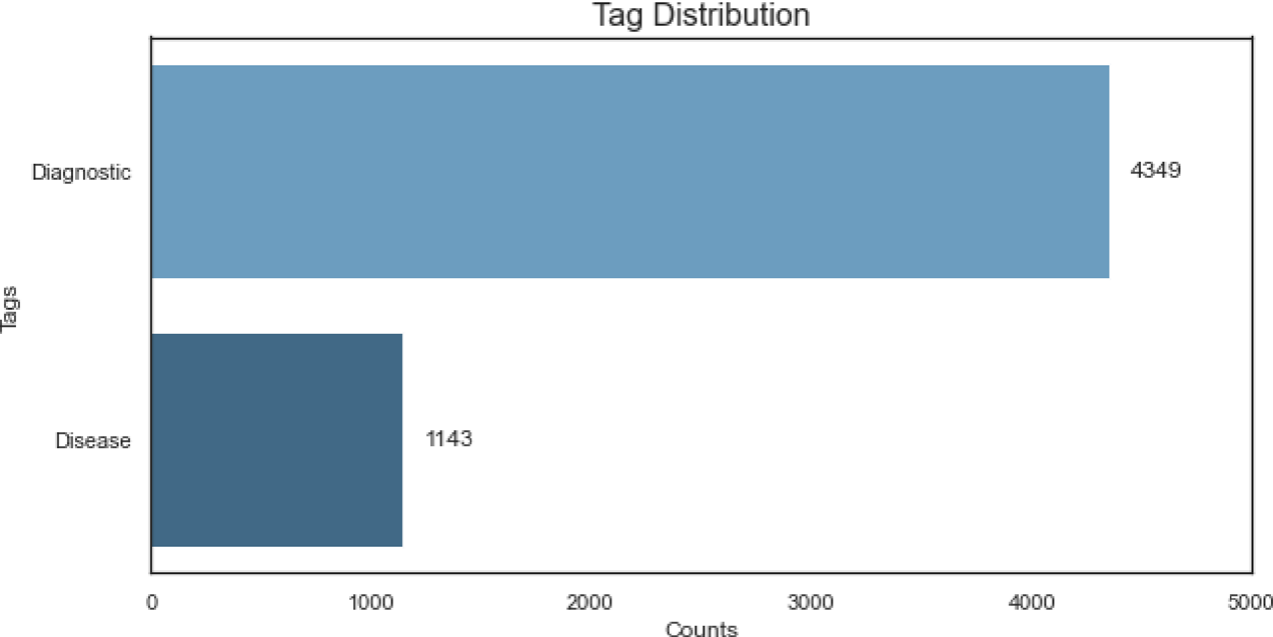
Tags distribution.

Following the manual annotation, an exploratory analysis of the counts per label under study was conducted, with a particular focus on the labels that identify the beginning of the entities (B-D and B-R). This approach facilitates a more discernible visualisation of the distribution and frequency of the primary entities under study, as illustrated in Fig. 5. The intermediate labels (I-D and I-R) were excluded from this figure to enhance clarity and simplicity, emphasising the initial positions of the entities, which are pivotal for comprehending the structure and initiation points of the annotations.

These steps guaranteed the data’s cleanliness, completeness and organisation for the application of language models, thereby facilitating analysis and obtaining reliable results. Accuracy at this stage was paramount for the effectiveness of the PTMs applied in the subsequent phases of the project.

## Modelling and evaluation

The objective of the modelling phase of this study was to perform fine-tuning on the PTMs using the annotated data, thereby enabling them to accurately classify the entities under study. Fine-tuning was chosen because PTMs are already pretrinaed on large datasets, capturing a wide range of language patterns^16^.

The selection of models was guided by the findings presented in the literature review. This process took into account previously identified challenges, including the nature of the data, the language used in the reports, the scale of the dataset and the specific outcomes sought.

The dataset comprised 305 annotated medical reports, with a total of 7,7309 tokens, giving an average of 253 tokens for each report.

To evaluate the performance of the selected models, a series of performance metrics were employed, including precision, F1-score, recall and accuracy^71^. Additionally, to gain a more nuanced understanding of how each model classified the entities, a detailed analysis was conducted using confusion matrices^71^.

Based on the literature review, the primary models selected were Albertina^52^, BERT^72^, and RoBERTa^73^, due to their relevance to the study’s objectives. Since the dataset consisted of Portuguese text in a cardiological context, these models were adapted to capture the linguistic and contextual nuances of the data.

In terms of accessibility and practicality, models from HuggingFace were selected, as they were readily available and did not require extensive parameterisation. Consequently, models such as BrainNERD^75^ (identified in the literature review), though potentially relevant, were excluded due to their limited availability.

### Modelling

In this phase, following the research methodology, the focus is on fine-tuning the PTMs to achieve the outlined objectives. It is essential to explain the rationale behind the chosen models and any parameter adjustments made during the fine-tuning process.

#### Selection of PTMs

First of all, Albertina^52^, which was advantageous due to its compatibility with the language of the dataset, its proven success in similar studies and its accessibility.

A model developed by the ISTAR-Iscte research group, MediAlbertina^53^ is a derivative of Albertina^52^ and belongs to the BERT^72^ family of encoders. This model, adapted from Albertina PT-PT 900^52^, has been designed for the specific purpose of performing NER tasks involving a range of entities, including diagnose, symptom, vital sign, outcome, medical procedure, medication, dosage and progress. These entities are directly relevant to the subject matter of this study.

Furthermore, the BERT^72^ family of transformers was then evaluated, given their established strong performance in contexts similar to this. Of particular interest within this family were BERTimbau^56^ and RoBERTa^73^. Furthermore, BlueBERT^50^ was included in the assessment as it has been effectively utilised in analogous contexts, thereby substantiating its relevance.

To keep track of the models used in training, Table 6 was created, listing the model names as they appear on Hugging Face.

**Table 6:** PTMs.

Model	Hugging face name model
BerTimbau	neuralmind/bert-large-portuguese-cased
Blue Bert	bionlp/bluebert_pubmed_mimic_uncased_L-12_H-768_A-12
Albertina 900	PORTULAN/albertina-900 m-portuguese-ptpt-encoder
Albertina 1500	PORTULAN/albertina-1b5-portuguese-ptpt-encoder
MediAlbertina 900	portugueseNLP/medialbertina_pt-pt_900m
MediAlbertina 1500	portugueseNLP/medialbertina_pt-pt_1.5b
Roberta	UCSD-VA-health/RadBERT-RoBERTa-4 m

#### Fine-tuning of PTMs

The process commences with the loading of a pre-trained model and a tokenizer for the NER task, in addition to the configuration of the correspondences between labels and indexes. Subsequently, the data is loaded, extracted and prepared for tokenization. To address the serialisation of NumPy objects to JSON, a custom class, NpEncoder^75^ is defined. Additionally, a function was implemented to calculate evaluation metrics, with these metrics being stored in a JSON file. The metrics are calculated and recorded at each epoch, allowing for continuous assessment of the model’s performance.

For fine-tuning the pre-trained models, specific training parameters were set: a learning rate of 0.00001 training for five epochs, a training batch size of 32, an evaluation batch size of 64, and one thousand warmup steps. Checkpoints were saved at regular intervals to capture the model’s progress and allow for resuming training if needed. This approach ensured that the fine-tuning process was efficient and that the model could be effectively optimised for the specific context of the study.

The computational demands for fine-tuning required substantial processing power. The models were trained on an Apple M2 Max with 64 GB of RAM, with an average runtime of five thousand seconds per model.

#### Performance metrics

The evaluation of the effectiveness of these PTMs and the assurance of their capacity to identify entities hinge upon the using of performance metrics.

During the fine-tuning process, a variety of metrics are monitored, including training loss, validation loss and accuracy. Training loss indicates the model’s error on training data, while validation loss evaluates performance on previously unseen data, allowing for the assessment of the model’s generalisation capabilities and the potential for overfitting. A minimal discrepancy between these two losses suggests an optimal model fit.

Accuracy is a measure of the proportion of correct predictions concerning the total number of predictions made by the model, providing an overview of its performance.

Furthermore, an analysis of the precision, recall and F1-score^71^ are analysed for each identified class and each fine-tuning epoch. However, it is important to note that these metrics are presented as macro averages to avoid bias from the entity “O”, which has the highest representativity and could skew the results if treated individually. Macro averages calculate the metrics for each class independently and then average them, ensuring a balanced evaluation across all classes. A more comprehensive evaluation was conducted using confusion matrix^50,71^, which demonstrated the distribution of accurate and inaccurate predictions in relation to the actual classes. These analyses facilitated an understanding of the model’s entity classification capabilities.

### Evaluation

In this phase of CRIPS-DM, the objective is to assess the quality and efficacy of the PTMs in the preceding modelling phase. This entails verifying that the models are indeed capable of solving the proposed problem and meeting the success criteria that were previously established.

This section presents the performance results of the various language models evaluated in the study. The evaluation phase, following the CRISP-DM, requires monitoring of the training process. To ensure that the results progress as expected, potential issues such as overfitting or underfitting are identified and addressed from the outset. The results of the fine-tuning process for the models on the data set under examination are presented in the following section.

Table 7. Fine-tuning results for each model presents the fine-tuning results for each language model evaluated in this study. The table includes key performance metrics such as training loss, validation loss, accuracy, precision and recall across five training epochs.

**Table 7:** Fine-tuning results for each model.

Epoch	Training loss	Validation loss	Accuracy	Precision	Recall
BERTimbau model					
1	1.3911	0.9620	0.6204	0.2342	0.2316
2	0.8205	0.6731	0.7270	0.4732	0.3384
3	0.6157	0.5051	0.8044	0.7498	0.5243
4	0.4725	0.4058	0.8482	0.7551	0.7452
5	0.3745	0.3796	0.8589	0.8006	0.7921
Blue BERT model					
1	0.8940	0.5951	0.7709	0.5961	0.3971
2	0.4930	0.4022	0.8653	0.7698	0.7596
3	0.3249	0.3433	0.8979	0.8298	0.8179
4	0.2423	0.3420	0.9084	0.8250	0.8541
5	0.1962	0.3560	0.9106	0.8406	0.8553
Albertina 900 model					
1	0.7374	0.4803	0.8136	0.7365	0.7157
2	0.3998	0.3817	0.8933	0.8304	0.8254
3	0.2373	0.3357	0.9167	0.8606	0.8799
4	0.1441	0.3140	0.9377	0.8999	0.8778
5	0.1011	0.3362	0.9413	0.9281	0.9009
Albertina 1500 model					
1	0.7195	0.4894	0.8311	0.8475	0.8961
2	0.3933	0.3088	0.9027	0.9019	0.8792
3	0.2332	0.2733	0.9239	0.8949	0.9006
4	0.1476	0.2550	0.9353	0.9216	0.9032
5	0.1030	0.2700	0.9416	0.9283	0.9158
MediAlbertina 900 model					
1	0.7196	0.4519	0.8372	0.5341	0.3220
2	0.3475	0.3392	0.8962	0.7218	0.5245
3	0.1804	0.2822	0.9272	0.7634	0.6706
4	0.1233	0.3143	0.9374	0.7833	0.8112
5	0.1169	0.3130	0.9402	0.8600	0.8262
MediAlbertina 1500 model					
1	0.7231	0.4417	0.8258	0.7412	0.7487
2	0.3218	0.2721	0.9174	0.8786	0.8440
3	0.1775	0.2586	0.9404	0.9124	0.9033
4	0.1113	0.2440	0.9485	0.9172	0.9244
5	0.0672	0.2529	0.9504	0.9233	0.9255
Roberta model					
1	1.4956	1.1377	0.5205	0.2283	0.2184
2	0.9532	0.8336	0.6633	0.2613	0.2825
3	0.8198	0.7334	0.7144	0.2871	0.3133
4	0.7072	0.6200	0.7646	0.5249	0.3372
5	0.6250	0.5684	0.7803	0.5777	0.4157

BERTimbau^56^ is a derivation from BERT Brazilian Portuguese training. The model was created for evaluation in three NLP tasks: textual sentence similarity, textual link recognition and NER. The results showed that BERTimbau^56^ outperformed multilingual BERT^72^.

BlueBERT^50^, a specialized BERT^72^ model is refined on medical and clinical texts to enhance NLP tasks in the healthcare industry by leveraging medical language context and nuances. In particular, BlueBERT^50^ has been trained on extensive datasets according to article^56^, including a wide range of clinical notes.

Albertina 900 version^52^ is a specialised model based on BERT^72^, designed with the characteristics and linguistic nuances of PT-PT.

Albertina version 1500, an upgrade on the previous version, is the latest model from the BERT^72^ family, based on the transformer neural architecture and developed on the DeBERTa model, achieving the most competitive performance for this language^77^.

MediAlbertina 900^53^, from the BERT^72^ family, based on DeBERTaV2^77^, resulting from the continuation of the pretraining of PORTULAN’s Albertina models with Electronic Medical Records (EMR) from the same hospital as the case study. This model adapts the Albertina PT-PT 900 M to the Portuguese electronic medical domain using masked language modelling^52^.

MediAlbertina 1500^53^ was created by adapting the Albertina PT-PT 1.5B^78^ domain to real EMRs from the same hospital as the 900 model, also using masked language modelling.

RoBERTa^73^ is a model based on NER transforms, trained on data from the breast cancer domain. It achieved positive results^58^ on sequence annotation tasks in the medical domain and its ability to handle multilingual datasets.

**Fig. 6:**
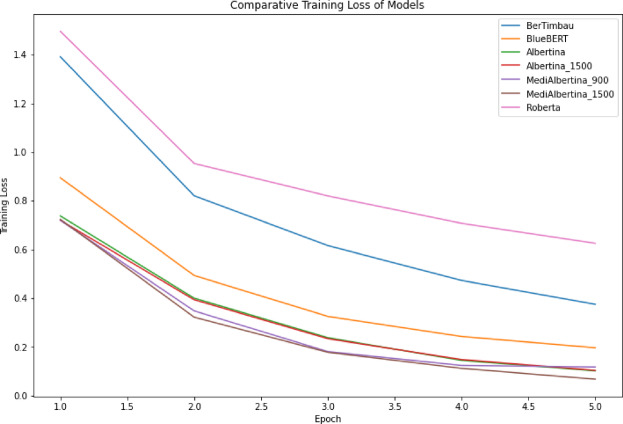
Training loss of models.

**Fig. 7:**
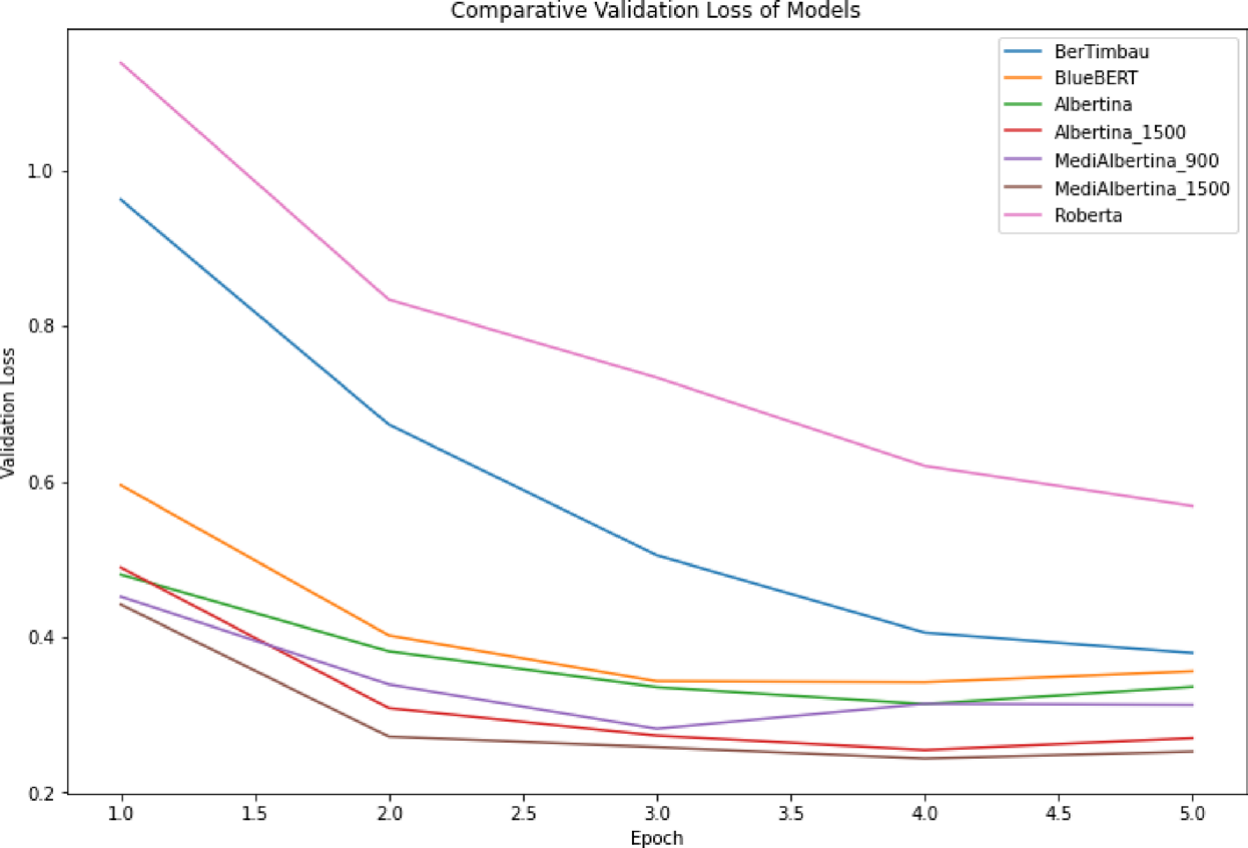
Validation loss of models.

**Fig. 8:**
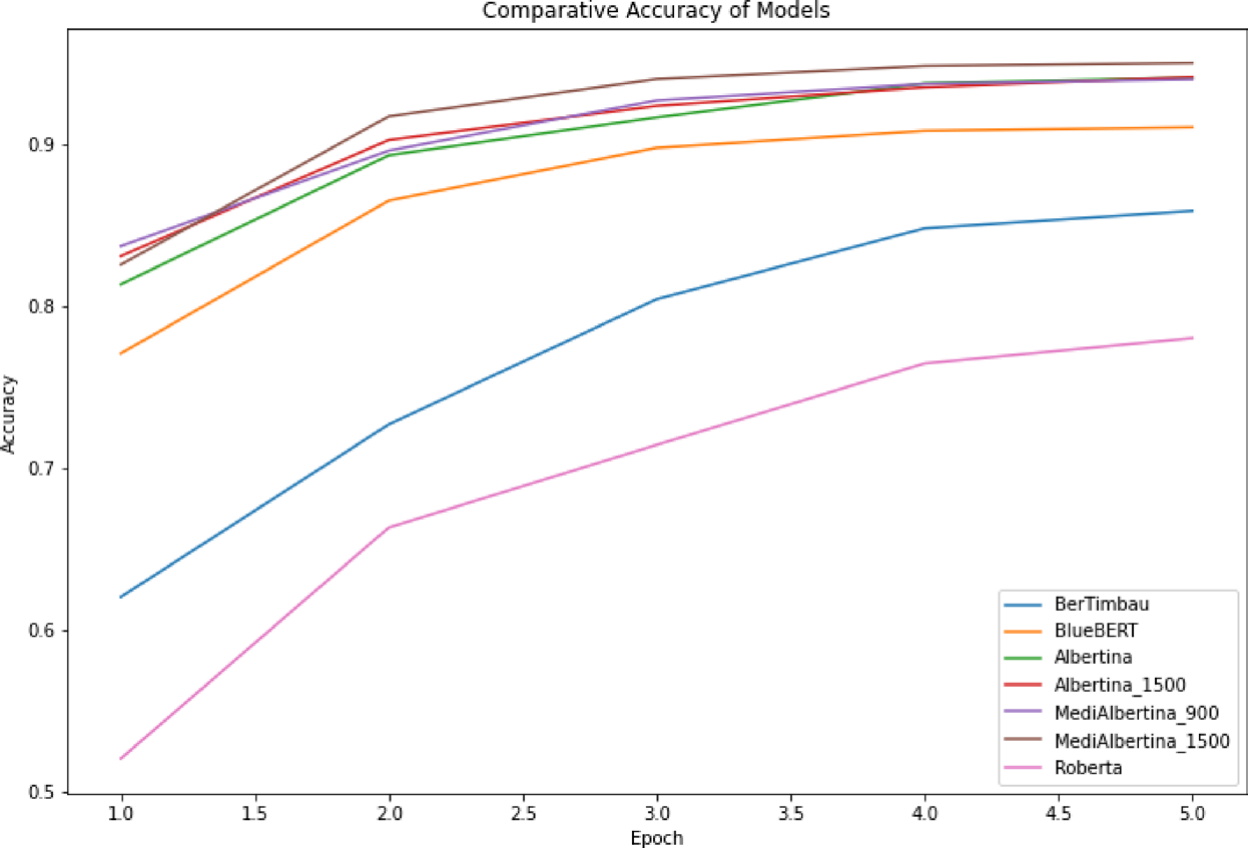
Accuracy of models.

The results are presented in a comparative visual format. Figure 6, depicts the evolution of training loss, which is expected to drop and stabilise when it reaches a good generalisation capacity. Figure 7 depicts the evolution of validation loss, which exhibits a similar behaviour to that of Fig. 6. Ideally, both training and validation losses should decrease and stabilise. A divergence, where training loss decreases significantly while validation loss does not, may indicate overfitting. Finally, Fig. 8 depicts the evolution of accuracy over the five fine-tuning epochs. The increase in accuracy should correlate with the decrease in both training and validation losses. If accuracy increases while validation loss decreases, it confirms that the model is learning effectively and generalising well to new data.

In general, the various models exhibited the anticipated trajectory during the fine-tuning phase. The Albertina models demonstrated a more promising performance, exhibiting consistently lower training and validation losses along with higher accuracy. This indicates superior learning and generalisation capabilities. This indicates that the Albertina architecture was well-suited to the specific data and task, potentially due to its capacity to capture intricate patterns and relationships within the dataset.

In contrast, the Roberta models exhibited a less favourable performance, with elevated training and validation losses and diminished accuracy compared to the Albertina models. This suggests that the Roberta architecture may have encountered challenges in effectively learning from the data, potentially due to a discrepancy between the model’s inductive biases and the characteristics of the dataset. Additionally, the Roberta models may have encountered issues such as overfitting or underfitting, which could have affected their capacity to generalise effectively to unseen data.

### Hyperparameters tuning

In the context of data mining, hyperparameter tuning involves adjusting the parameters governing PTMs to optimise their performance for a specific task or data set. This process differs from fine-tuning in that hyperparameter tuning aims to identify the optimal combination of parameters, including learning rate, batch size and number of epochs, to minimise validation loss and increase model accuracy^79^.

Considering the research process, the models exhibiting the most promising performance were identified for further tuning following the initial fine-tuning. A review was conducted of the steps taken, and it was determined that additional iteration should be undertaken on the models demonstrating the best performance and the lowest validation loss^80^.

The three best models identified in this way were MediAlbertina 1500, Albertina 1500 and Albertina 900. It was interesting to analyse the behaviour of the Albertina model in an older and a newer version and to understand the differences in model fit to the data, although the differences were small over five epochs.

In this hyperparameter tuning phase, the parameters that had the most significant impact on model performance were slightly increasing the learning rate, decreasing the batch size and increasing the number of epochs. Although increasing the number of epochs can help reduce validation loss and improve model fit, it also increases the risk of overfitting, so careful control of validation loss throughout this process was critical^81^. The results of the hyperparameter tuning are presented in Table 8 for the MediAlbertina 1500, Table 9 for the Albertina 1500 and Table 10 for the Albertina 900.

**Table 8:** Hyperparameter tuning results with the medialbertina 1500 model.

Epoch	Training loss	Validation loss	Accuracy	Precision	Recall
1	0.3279	0.3062	0.8924	0.8578	0.8023
2	0.2491	0.3086	0.9247	0.8778	0.8958
3	0.1610	0.2491	0.9428	0.9141	0.9185
4	0.1128	0.2829	0.9487	0.9141	0.9232
5	0.0854	0.2615	0.9474	0.9067	0.9301
6	0.0636	0.2389	0.9548	0.9252	0.9287
7	0.0511	0.2565	0.9538	0.9165	0.9334
8	0.0458	0.2619	0.9617	0.9383	0.9269
9	0.2800	0.2876	0.9611	0.9287	0.9316
10	0.0282	0.2956	0.9613	0.9257	0.9324

**Table 9:** Hyperparameter tuning results with the Albertina 900 model.

Epoch	Training loss	Validation loss	Accuracy	Precision	Recall
1	0.1146	0.3913	0.9245	0.8475	0.8961
2	0.1559	0.3130	0.9288	0.9019	0.8792
3	0.1206	0.2757	0.9369	0.8949	0.9006
4	0.1007	0.3131	0.9458	0.9216	0.9032
5	0.0795	0.3308	0.9532	0.9283	0.9158
6	0.0618	0.3681	0.9520	0.9263	0.9083
7	0.0514	0.3468	0.9554	0.9374	0.9126
8	0.0349	0.4485	0.9532	0.9203	0.9214
9	0.0293	0.3814	0.9532	0.931	0.9132
10	0.0240	0.3879	0.9543	0.9272	0.9214

**Table 10:** Hyperparameter tuning results with the Albertina 900 model.

Epoch	Training loss	Validation loss	Accuracy	Precision	Recall
1	0.1195	0.4992	0.9183	0.7365	0.7157
2	0.1592	0.3679	0.9345	0.8304	0.8254
3	0.1104	0.3829	0.9405	0.8606	0.8799
4	0.0919	0.4060	0.9473	0.8999	0.8778
5	0.0832	0.3581	0.9490	0.9281	0.9009
6	0.0696	0.3975	0.9489	0.9147	0.9179
7	0.0532	0.4020	0.9540	0.9291	0.9270
8	0.0401	0.4283	0.9569	0.9321	0.9192
9	0.0366	0.4288	0.9557	0.9330	0.9264
10	0.0289	0.4590	0.9562	0.9356	0.9274

**Fig. 9:**
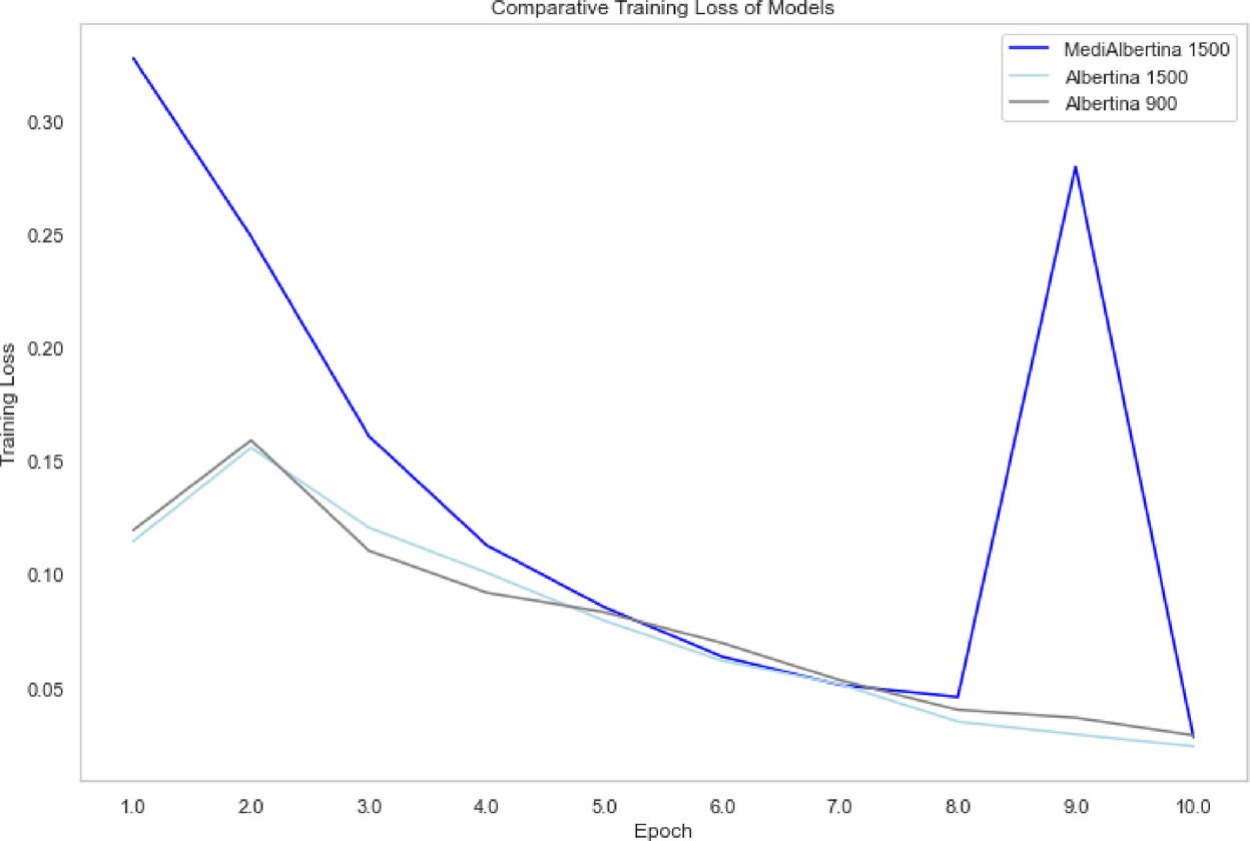
Training loss of hyperparameter tuning models.

**Fig. 10:**
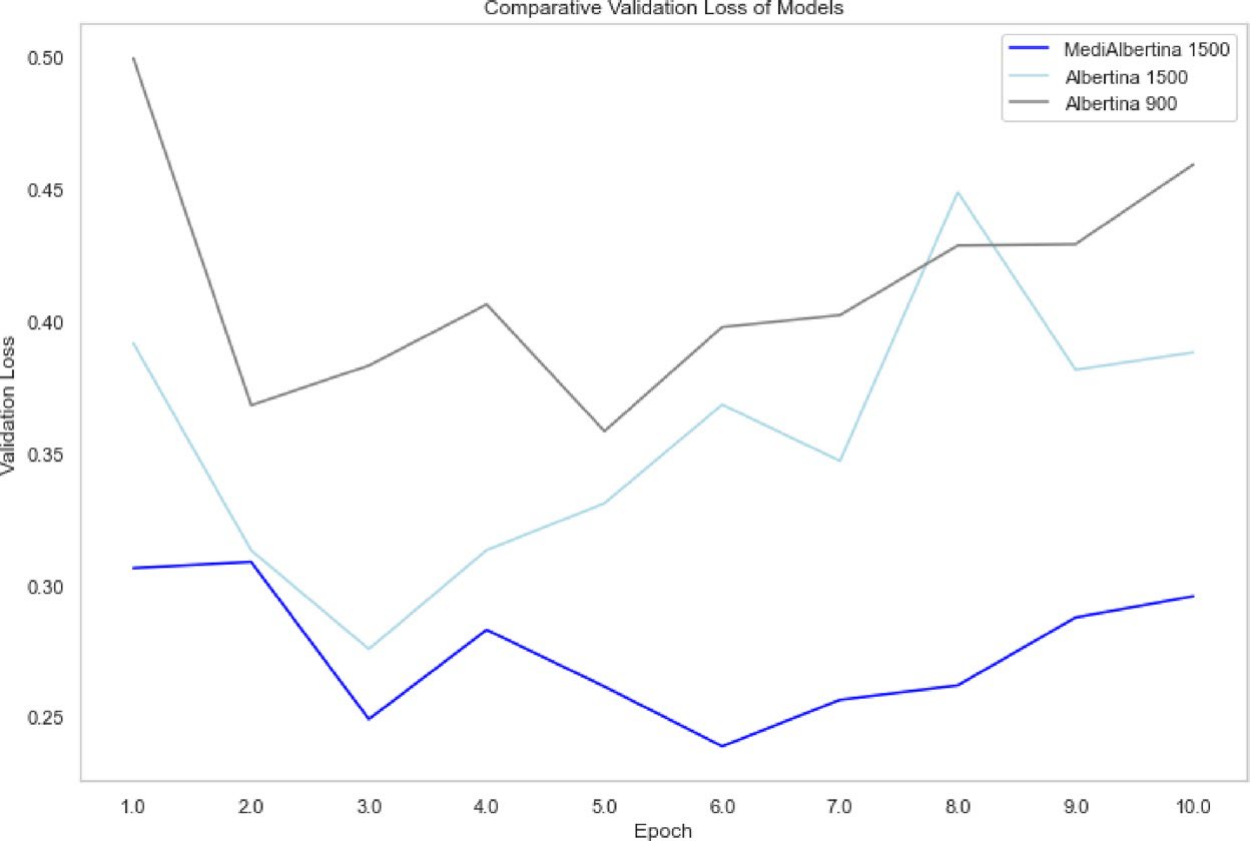
Validation loss of hyperparameter training models.

**Fig. 11:**
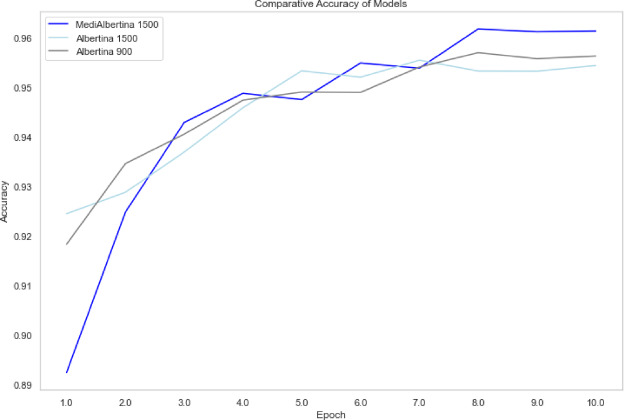
Accuracy of hyperparameter tuning models.

The evolution of the model’s performance is visually depicted in Fig. 9 for training loss, Fig. 10 for validation loss and Fig. 11 for accuracy. However, the training and validation loss curves do not exhibit the expected linear patterns typical in this type of analysis. Generally, the training loss curve should consistently decrease as the model better fits the data, while the validation loss curve should initially decrease, then stabilise, reflecting the model’s generalisation capability^82^. In this case, the curves only exhibit this expected behaviour up until epochs 8, 7 and 8 respectively, where the models achieved their highest accuracy.

The behaviour observed in the validation loss curves after epochs 8, 7 and 8 for the respective models suggests the onset of overfitting, where the models begin to memorise training data patterns rather than learning generalisable features. This is particularly evident in the divergence between decreasing training loss and increasing validation loss in later epochs. To mitigate this overfitting issue, several techniques could be implemented.

Early stopping represents a straightforward approach that would terminate training when validation loss begins to increase consistently, thereby preserving model generalisation ability. Based on the observations of this article, stopping at epochs 8, 7 and 8 for MediAlbertina 1500, Albertina 1500 and Albertina 900 respectively, would likely yield optimal results. Regularisation techniques such as L1 or L2 could also be beneficial by penalising large weights in the model, reducing complexity and improving generalisation. For transformer-based models such as the ones in the BERT family, dropout regularisation could also be adjusted to prevent co-adaptation of neurons.

Learning rate scheduling offers another promising strategy that could help fine-tune the optimisation process as training progresses, potentially stabilising validation loss in later epochs. Additionally, data augmentation methods, while challenging with medical text, could provide valuable improvements. Techniques such as synonym replacement or back-translation could provide additional training samples, helping the model learn more robust features.

The implementation of these techniques could potentially improve model generalisation and stability, particularly for MediAlbertina 1500, which showed the most promising performance metrics but also exhibited signs of overfitting after epoch 8. Future work should explore these mitigation strategies to further enhance model performance and reliability in clinical applications.

To assess the models’ performance, the average performance metrics from the final epoch, as shown in Table 11, were analysed. The MediAlbertina 1500 outperformed the other models across all evaluated metrics. It achieved a strong average performance, with 96.13% in accuracy, recall and F1-score. This high F1-score indicates that the model effectively balanced precision and recall, accurately classifying true positives with minimal trade-offs. Consequently, MediAlbertina 1500 demonstrated robust overall performance.

**Table 11:** Average weight of hyperparameter tuning models’ performance.

Model	Precision	Recall	F1-score
MediAlbertina 1500	0.9613	0.9613	0.9613
Albertina 1500	0.9542	0.9543	0.9542
Albertina 900	0.9566	0.9562	0.9563

The three models demonstrated satisfactory performance outcomes. However, it is also essential to consider the time investment required for training each model. As illustrated in Table 12, the Albertina 1500 model required the greatest amount of time for training, with a duration of 6.15 h. In contrast, the Albertina 900 model, which is less recent, exhibited the shortest training time among the analysed models, with a duration of 2.92 h. A reduction in training time is advantageous when time is limited, as evidenced by the favourable results. Conversely, a model requiring a lengthy training period may not be the most efficient option, given the necessity for substantial computational resources.

**Table 12:** **H**yperparameter tuning model train’s runtime.

Model	Train runtime (seconds)	Train runtime (hours)
MediAlbertina 1500	18,859.3731	5.2387
Albertina 1500	22,153.3407	6.1537
Albertina 900	10,527.4043	2.9243

**Fig. 12:**
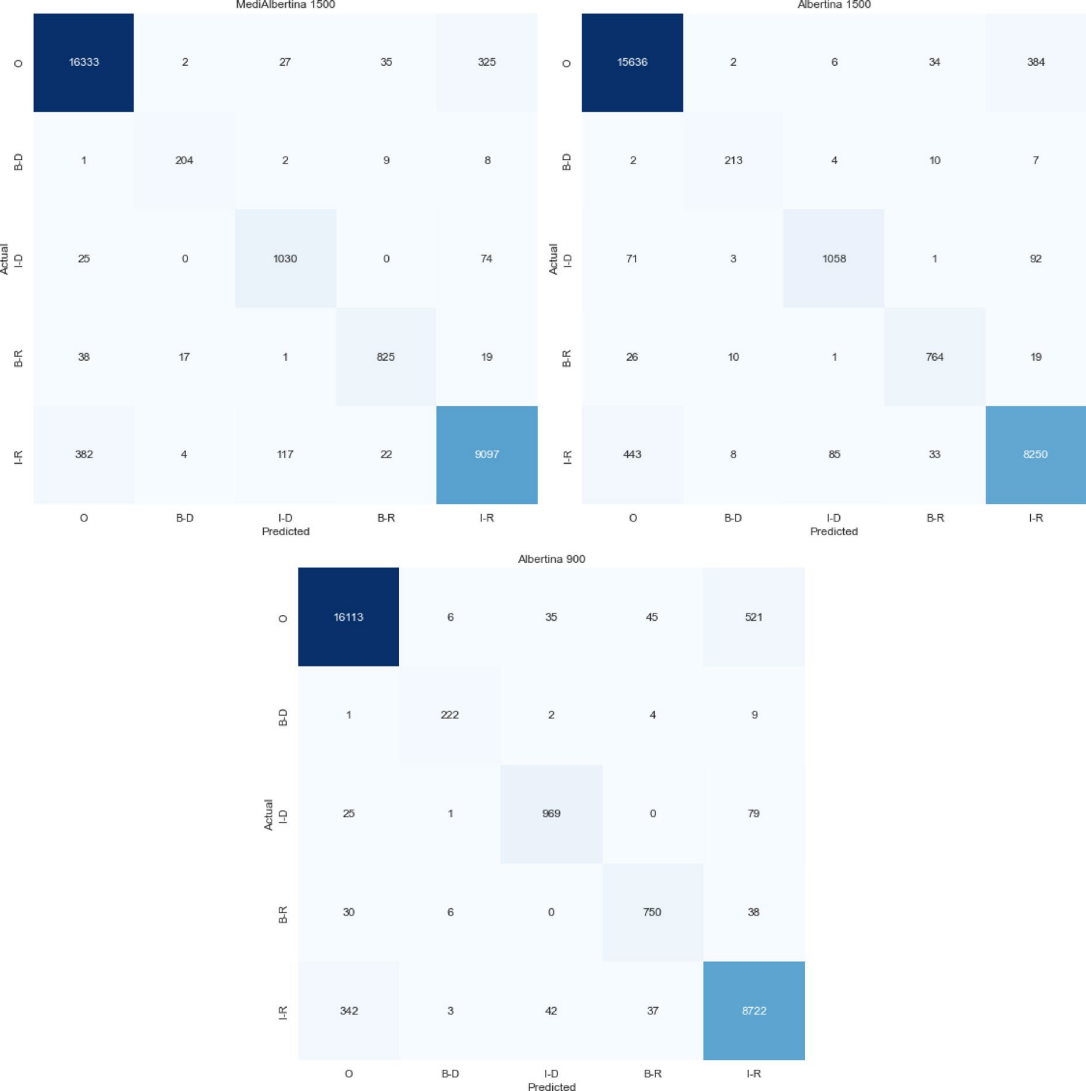
Confusion matrix of hyperparameter tuning models.

A review of the confusion matrix for the models, as illustrated in Fig. 12, reveals that MediAlbertina 1500 exhibits superior precision in comparison to the other models, consistent with the previous fine-tuning. Overall, the entities were more accurately classified than in previous instances. The ‘O’ class continues to be the most precisely classified.

Following the identification of the three models with the most promising initial performance: MediAlbertina 1500 with an accuracy of 95%, followed by Albertina 1500 and Albertina 900, each with an accuracy of 94.1%, the subsequent phase involved the application of hyperparameter tuning.

This process entailed the refinement of key parameters, including the learning rate, batch size and number of epochs, relative to the previous iteration. The results of these adjustments were evident in the enhanced performance of the models, with MediAlbertina 1500 achieving an accuracy of 96.2%, Albertina 1500 reaching 95.5% and Albertina 900 attaining 95.7%. These improvements represent an overall increase of one to two% points.

## Deployment

**Fig. 13:**
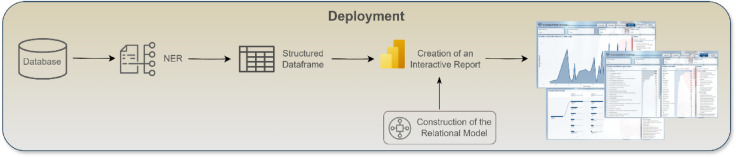
Identified final steps to achieve the identified goals in this research.

The deployment phase of this research is illustrated in Fig. 13 which, similar to Fig. 3, illustrates the research workflow, this time focusing on the final phase of the study design. During this phase, the model processes cardiology reports derived from medical imaging exams, extracting critical entities such as “disease” and “diagnosis” and organising them into a structured data format. This structured data is subsequently utilised to generate an interactive report in Power BI, offering a user-friendly interface that aids physicians in the screening process and, consequently, in their decision-making. The intuitive nature of Power BI’s interface ensures that the resulting reports are readily interpretable, enabling healthcare professionals to swiftly extract the most relevant information and respond accordingly. The outcome of this implementation serves as a proof of concept, illustrating the model’s potential for deployment in real-world clinical environments, such as hospitals.

Therefore, the final phase of the CRISP-DM methodology, designated as deployment, was pivotal in transforming the findings of this research into practical and useful tools for health professionals. In particular, the results were presented through a dynamic report developed in Power BI, which allows for the interactive and detailed visualisation of the information extracted from the survey of diseases and diagnosis, as derived from the analysed reports. This approach is more efficient than the manual screening currently employed, whereby the average time required to analyse each report is approximately seven minutes.

The tool developed in this study enables the extraction of essential information from text reports, including the mapping of identified diseases, which can be correlated with the type of examination performed or the patient’s age, as well as the identified diagnoses.

After testing several models, the MediAlbertina 1500 model demonstrated the most promising performance in identifying and classifying medical conditions from the text of these reports. Based on its results, the outputs from this model were used to create an accessible and interactive report aimed at assisting healthcare professionals in analysing the extracted data more effectively.

The report was developed using Power BI, chosen for its ability to create dynamic and interactive reports that present complex medical data in a clear, intuitive format. This interactivity allows healthcare professionals to explore the data more efficiently, facilitating faster and better-informed decision-making.

The report is based on data that NER has transformed. NER has organised this data into a structured data frame and exported it in CSV format. In sequence, this CSV file is used to feed the fact table that makes up the report. This includes the diseases identified and the corresponding diagnoses extracted from the medical imaging reports. The dimension tables provide supplementary context, enhancing the overall analytical framework on Table 13.

**Table 13:** Dimension table mapping.

Dimension table	Describe
Dim_validation_timestamp	Tracks the time when the data was validated
Dim_exam_type	Categorises the types of exams performed
Dim_patient_birthday	Tracks patients’ date of birth
Dim_disease	Lists the identified diseases

The report was structured into distinct analytical sections, each devoted to a particular topic.

**Fig. 14:**
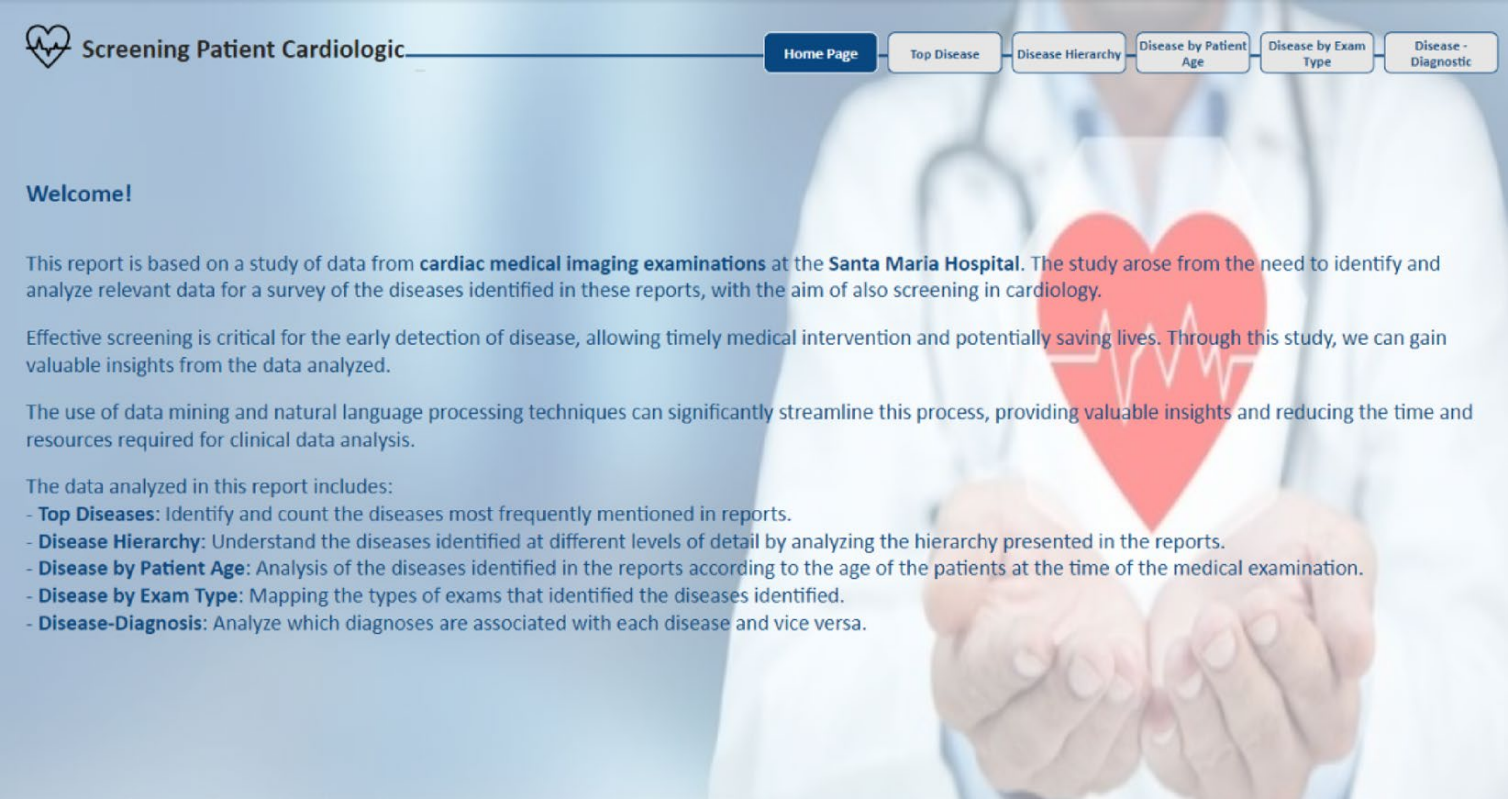
Homereport page.

The presentation commenced with an overview of the report, “Home Page” as in Fig. 14. Homereport page, elucidating its context, contribution and purpose. It also delineated the source of the data, namely cardiac medical imaging examinations from Hospital de Santa Maria and underscored the significance of screening for this cardiology department.

**Fig. 15:**
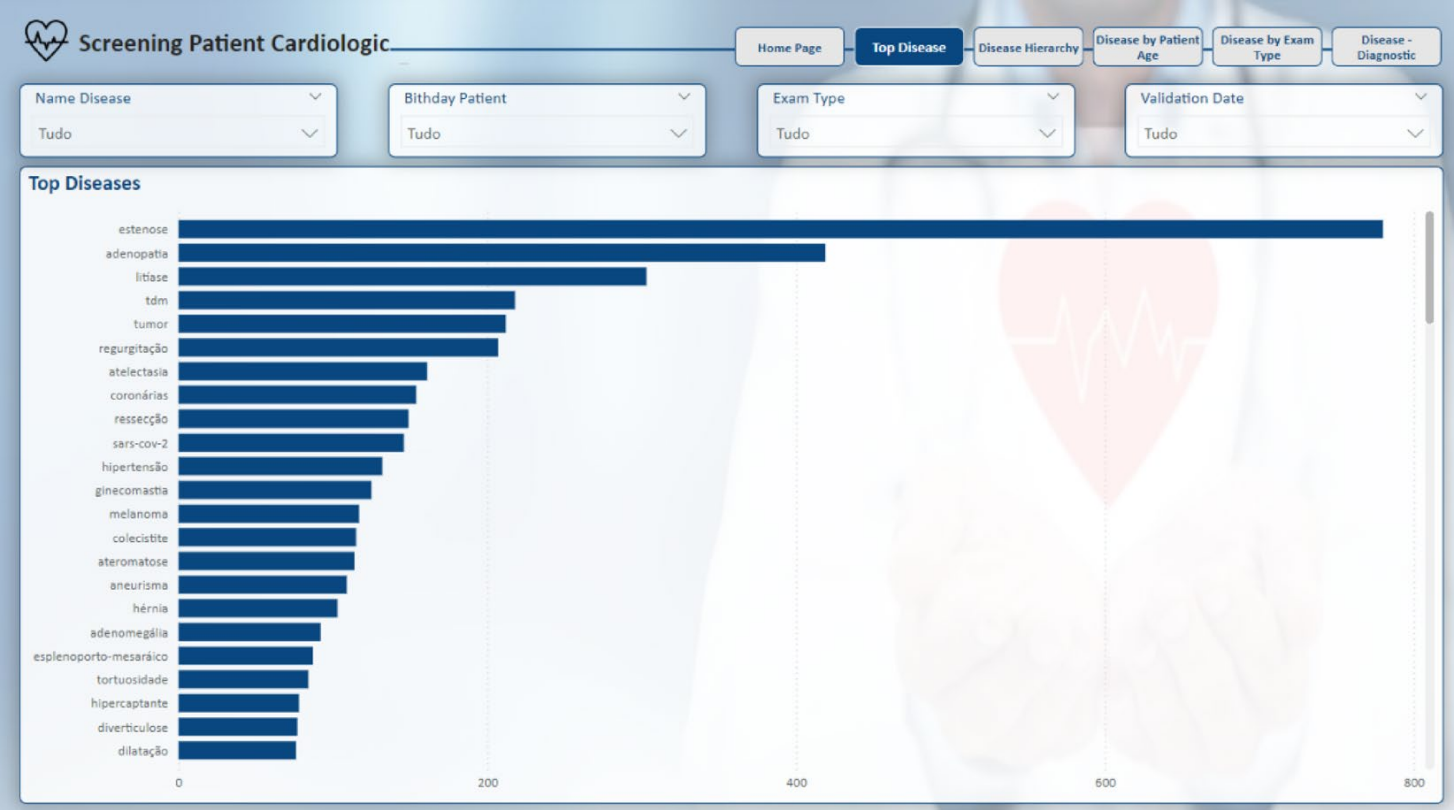
Top disease report page.

**Fig. 16:**
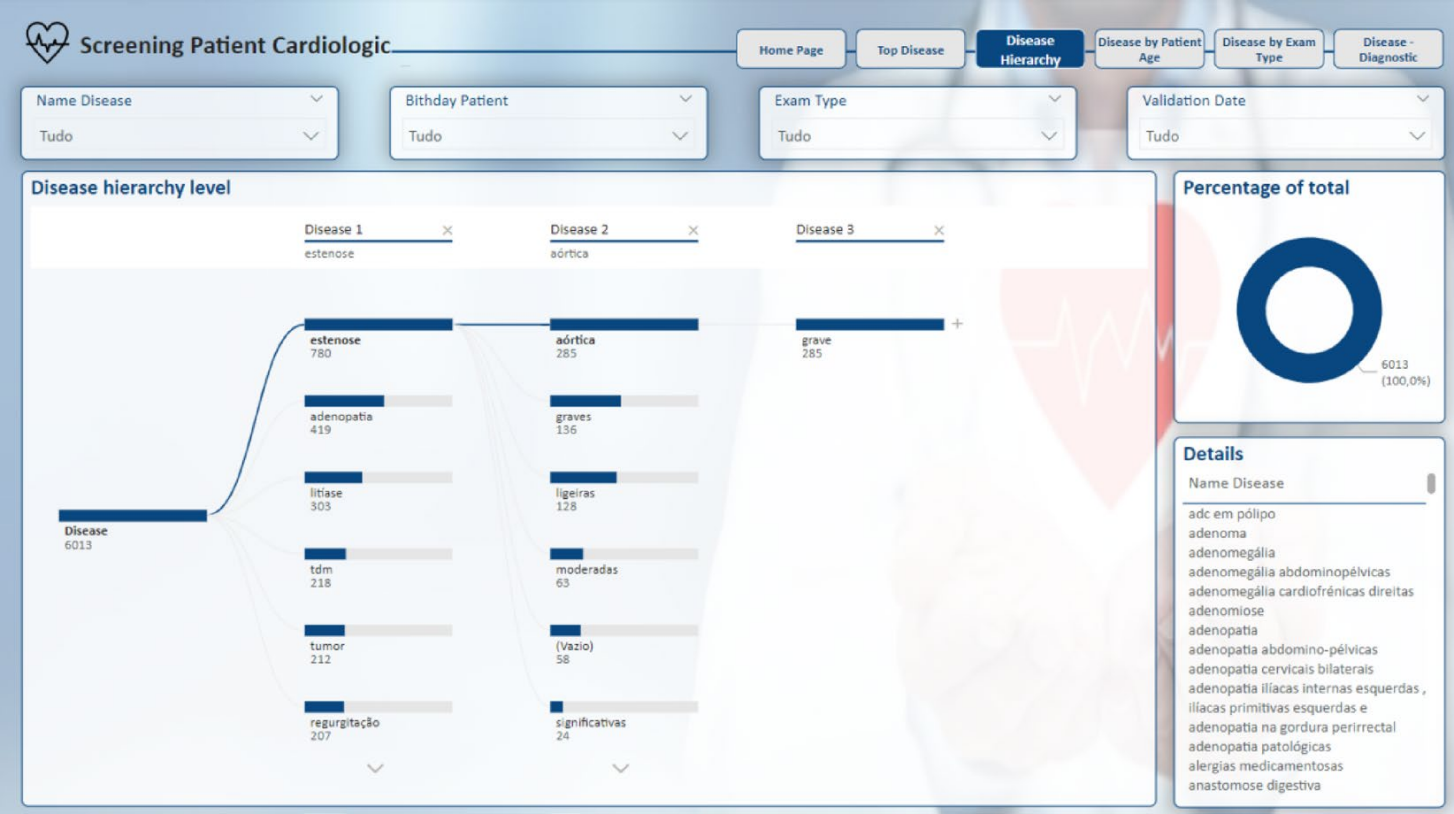
Disease hierarchy report page.

The initial analysis page, shown in Fig. 15 and titled “Top Diseases,” serves as a key tool for screening the diseases identified in the medical reports. This page provides an overview of the most frequently mentioned conditions across the dataset, allowing users to grasp the primary diseases under consideration quickly (Fig 16).

Subsequent pages present filters that can be applied to refine the analysis, such as “Name Disease”, “Birthday Patient”, “Exam Type” and “Validation Date.” These filters enable users to focus on specific aspects of the data, such as narrowing the analysis to a particular age group or a specific type of examination, depending on their needs.

**Fig. 17:**
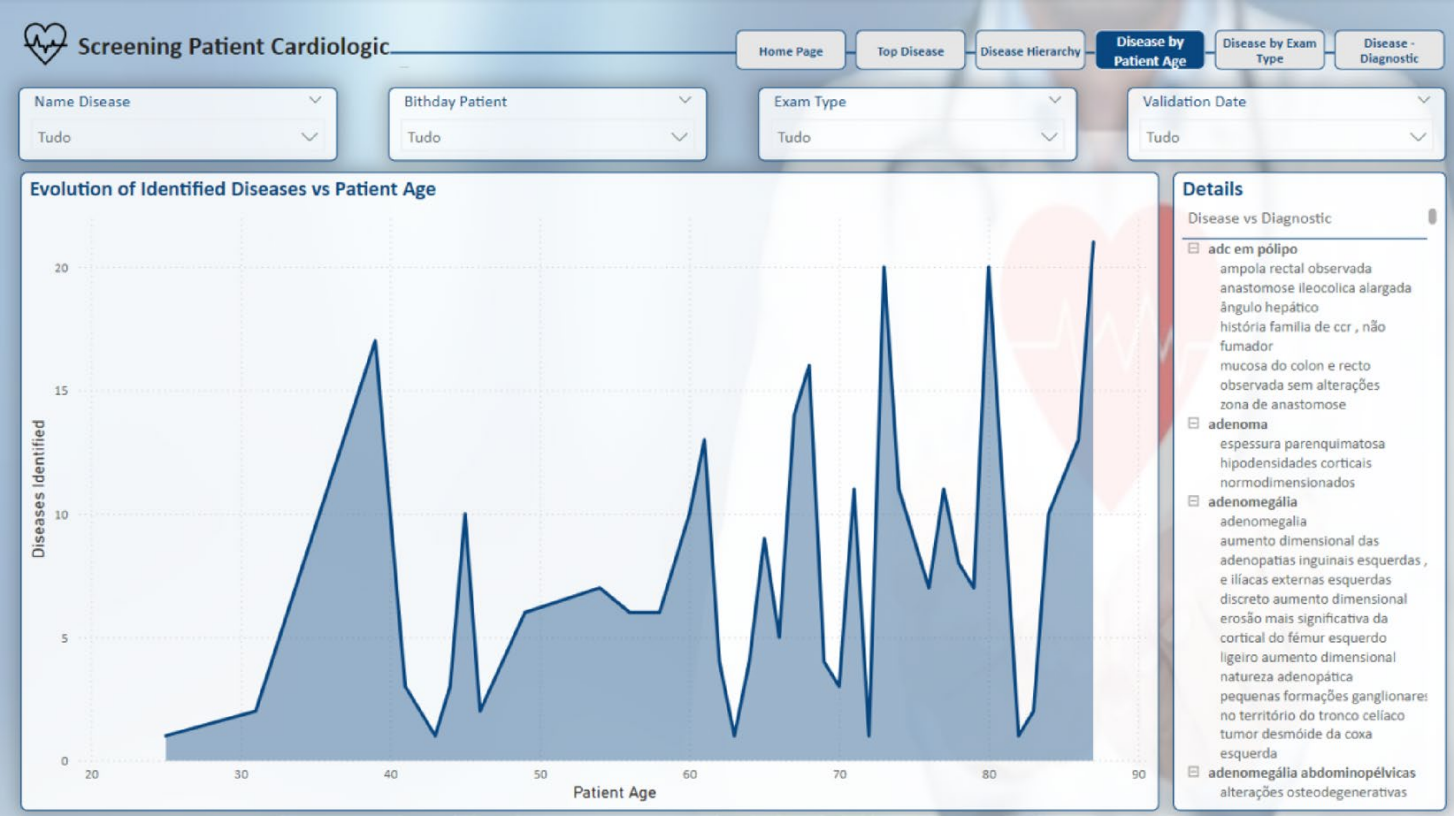
Disease by patient age report page.

The “Top Diseases” analysis in Fig. 15 highlights the diseases that appear most often in the reports, offering a straightforward way to screen which conditions are prevalent in the dataset. The “Top Diseases” analysis in Fig. 17 highlights the diseases that appear most frequently in the reports, providing a clear overview of the most prevalent conditions in the dataset. While the presented data excludes patient identifiers to ensure the protection of sensitive information, the hospital’s operational database includes this information. This allows for the tracking and matching of patients via unique IDs when necessary for clinical or administrative purposes, ensuring that the analysis can be tied back to individual cases within a controlled and secure environment.

This functionality is useful for gaining an initial understanding of the distribution of diseases within the context under investigation, helping users to explore and interpret the data efficiently.

The subsequent page retains the structural consistency of the previous ones. The “Disease Hierarchy” page depicted in Fig. 16 offers an in-depth exploration of the diseases identified in the reports. This page provides a clear visualisation of the hierarchical structure of these diseases, breaking them down into various levels of detail.

The term “disease” can be disaggregated into multiple levels based on the entities classified under this term. For instance, if a disease name comprises four words, the model identifies these sequentially as “B-D,” “I-D,” “I-D,” and “I-D.” These components are then aggregated according to their level of detail, starting with the broadest category and narrowing down to the most specific. This hierarchical structure also allows for the grouping of diseases from different domains that share a common prefix, thereby facilitating a more nuanced understanding of how different diseases are categorised and related.

Furthermore, the analysis can be refined by considering the patient’s age, as illustrated on the “Disease by Patient Age” page, Fig. 17. This permits a stratified analysis by patient age, wherein a line graph illustrates the distribution of identified diseases across different age groups. Adjacent to the graph, a detailed description is provided, correlating the diseases with the respective diagnoses identified. For a more targeted analysis, users can apply filters to focus on a specific disease, allowing for a more in-depth exploration of its prevalence in various age groups, as well as the diagnoses associated with those specific cases.

**Fig. 18:**
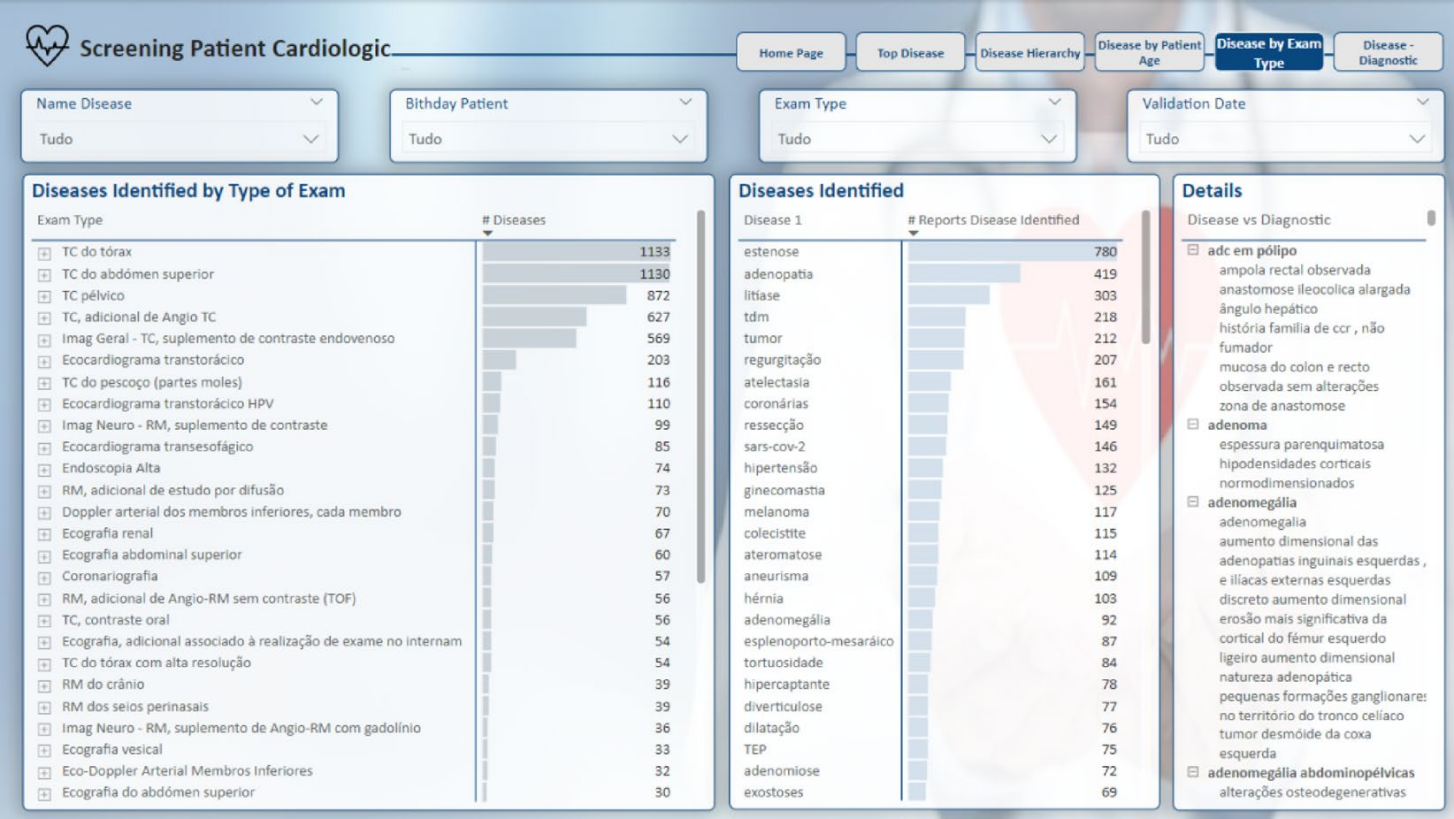
Disease by exam type report page.

Similarly, Fig. 18 depicts the “Disease by Exam Type” page, which adheres to the same structural framework as the preceding pages. Its objective is to facilitate the mapping of exam types that have identified diseases, thereby enhancing comprehension of the efficacy of diverse exams in detecting specific diseases. A matrix is presented, displaying aggregated dimensions, specifically “Exam Type” and diseases.

This aggregation enables the identification of the diseases most frequently identified by type of exam. Additionally, a list of the most identified diseases is provided, along with details of the diseases versus diagnoses, as seen on previous pages. The objective is to enhance the study with additional dimensions, enabling the screening of specific types of exams. By selecting this option in the initial matrix, the remaining elements can be filtered out, and the identified diseases, along with their respective diagnoses for this particular type of exam can be viewed.

**Fig. 19:**
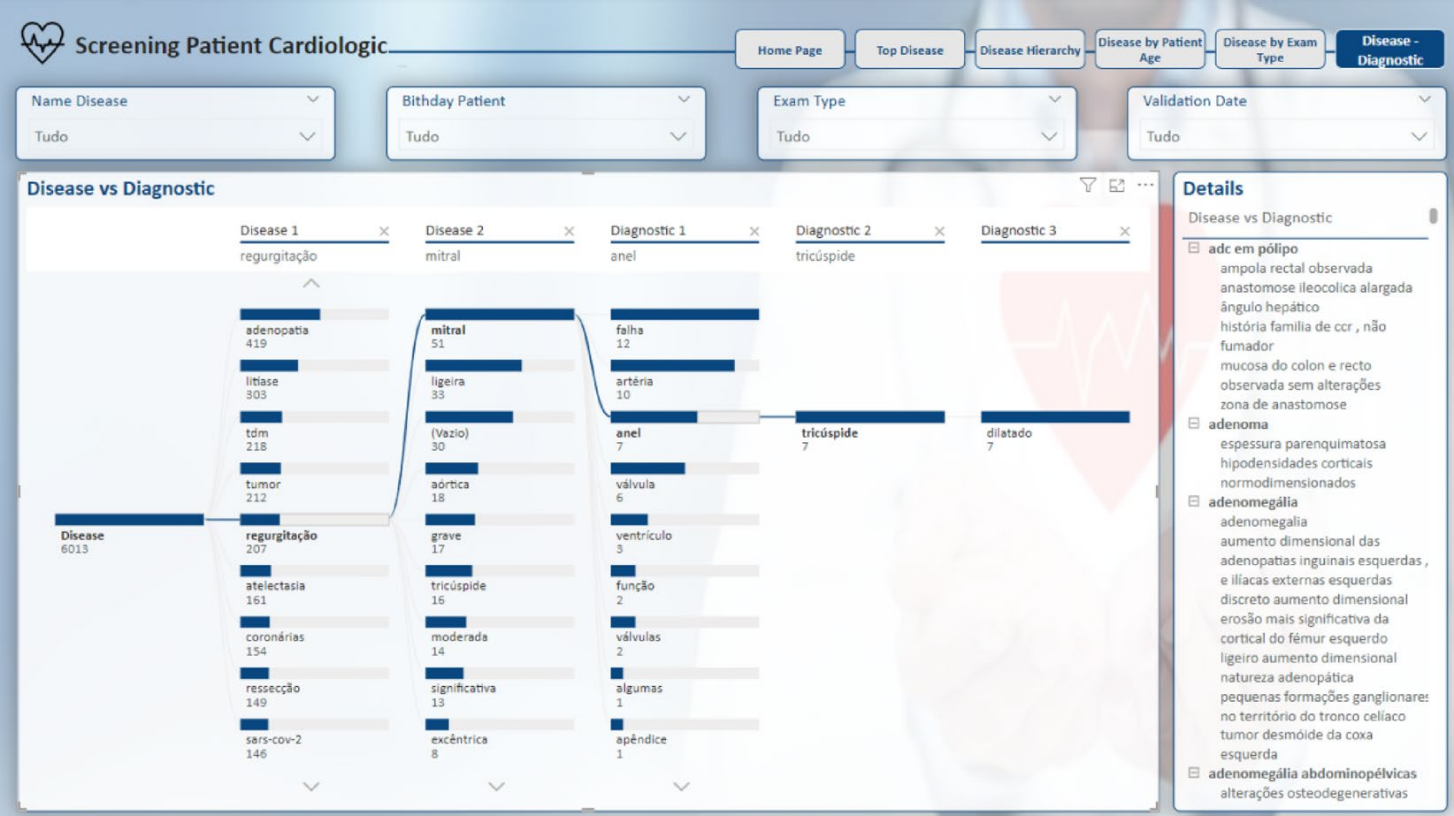
Disease - diagnostic report page.

Finally, the “Disease-Diagnosis” page, depicted in Fig. 19, allows for a detailed analysis of the associations between diseases and diagnoses, offering a comprehensive view of the interrelationships between these clinical entities. This page serves the purpose of establishing clear links between diseases and diagnoses, providing a deeper understanding of the correlations at different levels of disease specificity. Through this disaggregation, it becomes possible to discern how the first and second levels of disease detail relate to specific diagnoses.

For example, Fig. 19 highlights the identification of the disease “regurgitation” on 207 occasions, of which 51 were specified as “mitral regurgitation.” Among these occurrences, “mitral regurgitation” was associated with the diagnosis of “dilated tricuspid ring” in seven cases. This type of analysis significantly facilitates screening, allowing healthcare professionals to efficiently identify clinical patterns and associations, thereby accelerating decision-making and identifying areas that may require further attention.

The analysis conducted in the report fulfils the initial objective of the screening investigation and provides a tool that facilitates the screening of cardiological diseases from medical imaging reports. This tool enables healthcare professionals to rapidly identify the most prevalent diseases, comprehend their distribution by age and examination type and investigate the relationships between diseases and diagnoses. This facilitates more expedient and efficacious medical interventions, conserving time and resources and potentially enhancing patient outcomes.

The visuals and the aforementioned pages serve merely as exemplars of the types of information that can be extracted from the reports. Additional data can be derived by analysing additional fields in similar reports or by supplementing them with data from other medical domains. The essential prerequisite for this is an identifier that links the patient to the relevant data. Once this is established, a multitude of associations can be made.

## Conclusion

### Discussion

**Fig. 20:**
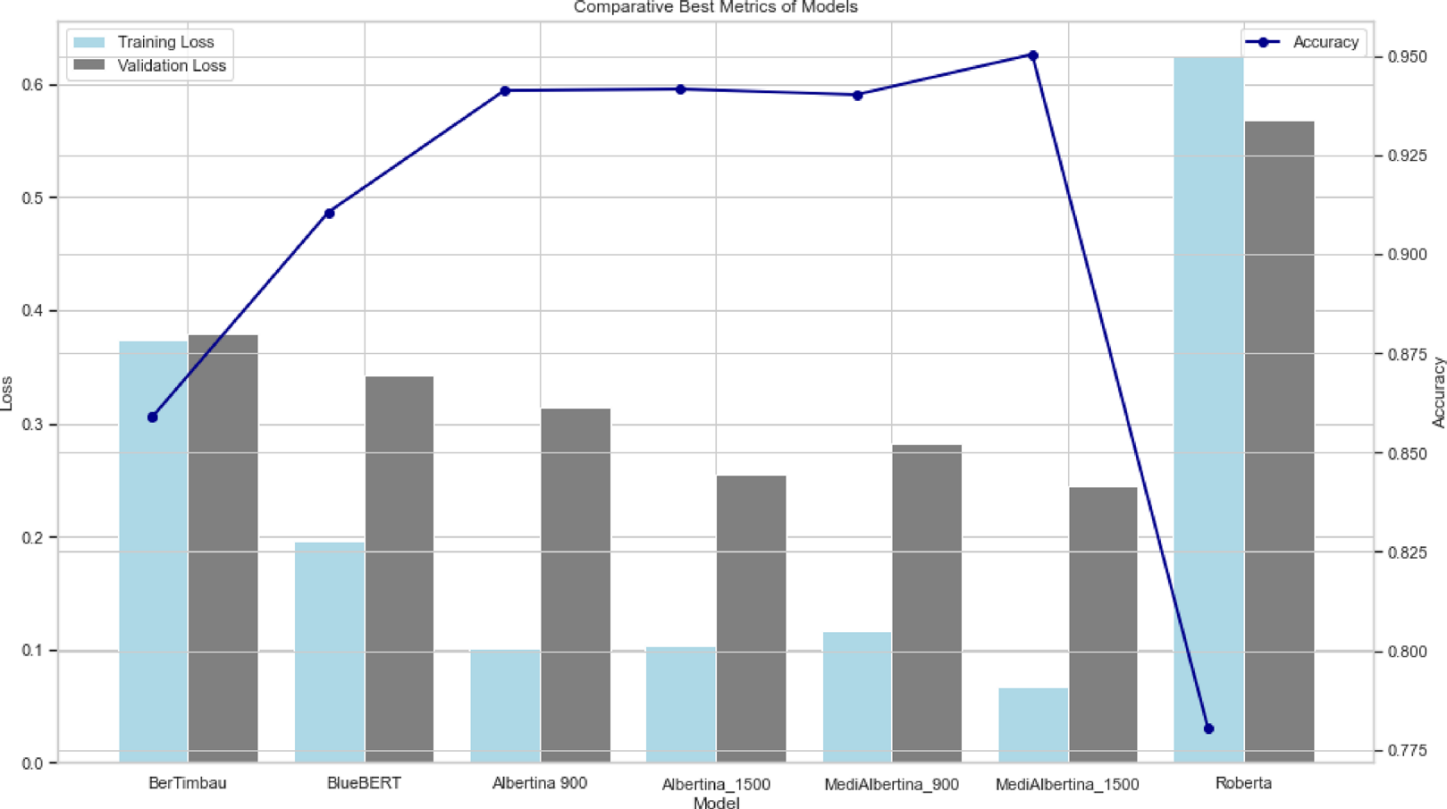
Visualisation of the best performance values of the different models.

Figure 20 demonstratively depicts the performances of the models under study. It was decided to present the best performance values of the models, typically from the last epoch, to ensure a fairer comparison.

Based on the results, the BERTimbau model performed robustly, achieving its maximum accuracy in the fifth epoch with 85.89%.Both training and validation loss tended to decrease, indicating a good fit of the model to the data.

Similarly, the BlueBERT model from the same family showed similar behaviour, but with higher accuracy values, reaching up to 91.06%.

Regarding the Albertina family models, the most promising performance was achieved by MediAlbertina 1500, with a maximum accuracy of 95.04% and a very low validation loss, indicating a good fit to the medical data under studied.

The Albertina 1500 followed closely with 94.02%, then Albertina 900 with 94.13%, and finally MediAlbertina 900 with 94.02%. The models exhibited minimal and uniform validation losses, indicating robust generalisation capabilities. This is likely attributable to their training on Portuguese vocabulary, particularly MediAlbertina, which was trained on clinical Portuguese vocabulary^83^.

As expected, the Roberta model proved to be the least efficient, with a maximum accuracy of 78.03%. It started with very high training and validation loss values, which eventually decreased. This inferior performance could be attributed to several factors^73^, such as the different domains of the data, as the model was trained on specific concepts of breast cancer, making it difficult to adapt to the new terminology and linguistic patterns.

In addition, although this model is based on the BERT architecture, it is a less recent version and therefore less advanced, which may also have affected its performance. Finally, suboptimal parameters during training may have contributed to this less efficient fit^84,85^.

In conclusion, the inferior performance of the RoBERTa^73^ model can be attributed to its domain specialisation, the outdated nature of its model version and the limitations of the initial fine-tuning process.

In contrast, the models of the Albertina family generally achieved high performance. Notably, the more recent versions, such as MediAlbertina 1500 and Albertina 1500, demonstrated significant improvements over their earlier versions (900).

This analysis is consistent with the macro-average performance metrics presented in the preceding tables, which have demonstrated a consistent evolution in performance over the epochs, with notable variations between the PTMs. MediAbertina_1500 and Abertina_1500 exhibited the highest precision and recall values, reaching an F1-score of over 0.92 in the final epochs. These outcomes suggest that both models demonstrate consistent and reliable performance, establishing them as the most effective in terms of overall performance.

In the middle range, the Albertina_900 is followed by the MediAbertina_900, but both models have demonstrated performance that is below the more robust models.

**Fig. 21:**
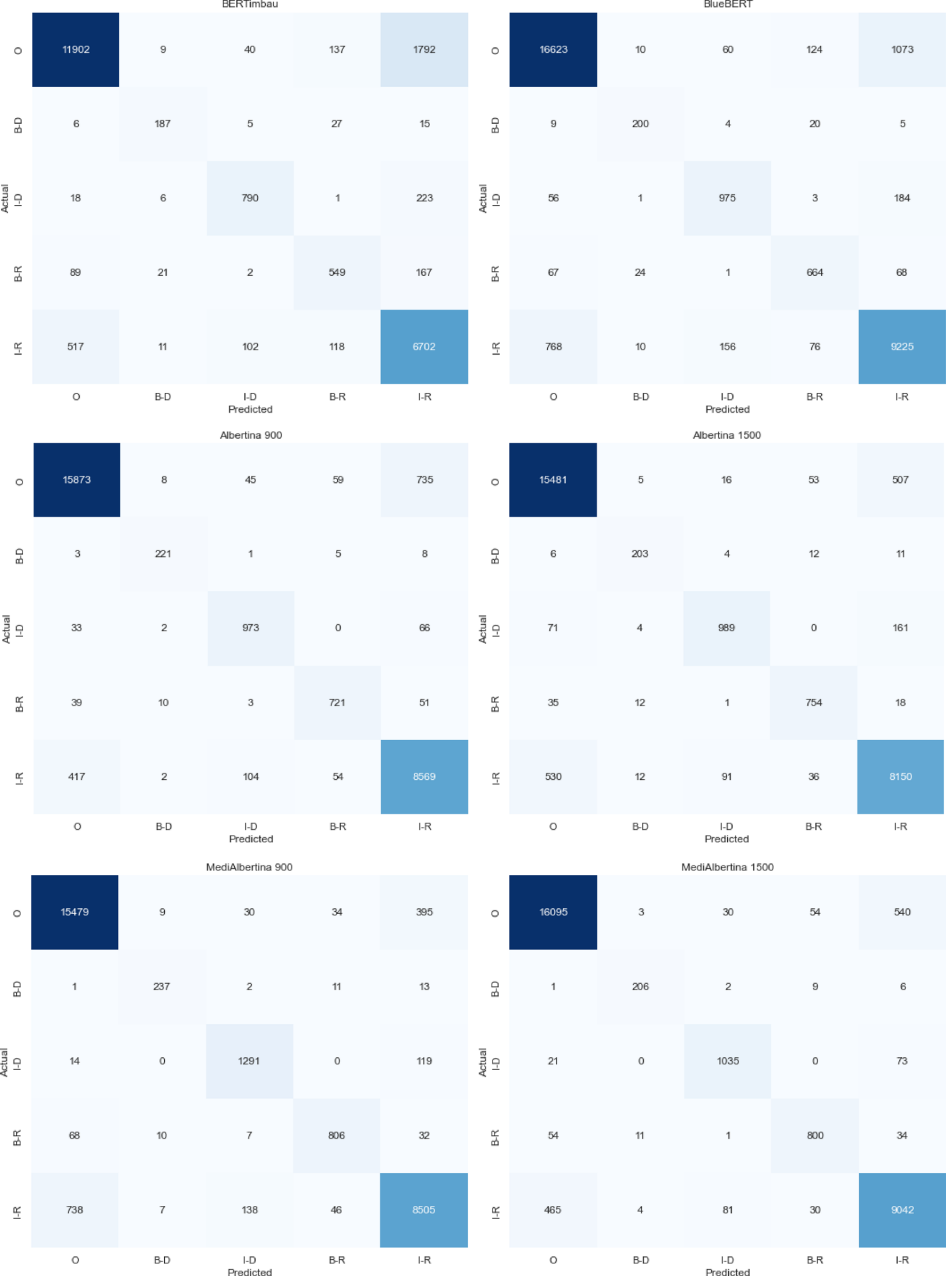
Confusion matrix of the models.

Regarding the confusion matrix values of the different models, as shown in Fig. 21, the analysis was conducted based on each model’s best-performing epoch, typically the final epoch. These matrices provide a detailed view of how each model classified the entities in the study data, specifically the categories “O”, “B-D”, “I-D”, “B-R”, and “I-R”^86^.

From this analysis, it can be concluded that the “O” class is consistently well classified in all models with high precision, always showing a high value. The most common error is its incorrect classification as “I-R”, and the opposite is also observed.

The high accuracy of the “O” class can be attributed to its overwhelming presence in the dataset, making it easier for models to learn and classify correctly. Since non-entity tokens vastly outnumber named entities, the models develop a strong bias toward correctly identifying them. Additionally, “O” tokens often appear in clear contextual separation from entity-related terms, reducing ambiguity. In contrast, the “I-R” class had the lowest performance values, likely due to its reliance on correctly classifying the preceding “B-R” token. If the beginning of a diagnosis-related entity is misclassified, the continuation token (“I-R”) is also likely to be incorrect. Furthermore, entity boundary ambiguity and lower representation in the dataset make distinguishing “I-R” tokens from non-entities more challenging. The confusion matrix (Fig. 10) reflects these challenges, showing frequent misclassifications between “O” and “I-R.” These patterns align with known difficulties in NER, where entity continuation tokens tend to have higher error rates due to their dependence on precise boundary detection and contextual cues.

Concerning the class representing a disease, denoted as “B-D” and “I-D”, it can be noted that the instances are rarely confused with one another, but in general they are well classified considering their proportions.

Finally, the classes representing diagnosis, “B-R” and “I-R,” are also well classified considering their total number, but they are often misclassified with the “O” class.

Overall, MediAlbertina 1500 and MediAlbertina 900 showed superior performance in terms of correctly classifying entities, with less confusion between entities compared to the other models.

### Final remarks

The research presented in this paper was prompted by the need for a more efficient approach to the surveying of diseases identified in medical reports in order to facilitate the screening process. This need was identified by a clinician in a hospital setting, specifically in the field of cardiology, given that the current manual process is notably time-consuming.

The current average time needed to analyse each medical report, invoked by this clinician, is approximately seven minutes. Out of the 12,651 reports included in this study, the total time required for reviewing them all is 1,475.95 h, which is about 61.5 days. This is equivalent to 210.85 working days. If a clinician dedicates three hours per day to this task, it would take 492 days (excluding vacation time) to complete. To achieve this within six months, the efforts of three clinicians would be required simultaneously. This substantial investment of time highlights the necessity for the implementation of a more efficient system.

Beyond the monetary savings, the system provides significant indirect benefits. By freeing clinicians from repetitive tasks, they gain valuable time to focus on more critical activities, such as seeing more patients, participating in research or contributing to other initiatives. This increased availability directly improves the quality of patient care by making medical processes more agile and responsive. Patients benefit from faster report turnaround times, leading to faster diagnoses and treatment plans that ultimately improve healthcare outcomes. In addition, the optimised allocation of medical resources helps hospitals operate more efficiently, amplifying the overall impact of this solution.

This study sought to address this challenge by employing NLP techniques to identify and classify medical entities, such as diseases and diagnoses, from unstructured text in cardiac medical reports. The ultimate aim was to streamline the identification and analysis process, thereby facilitating more efficient patient screening (G1) and enabling the establishment of relationships between diagnoses and diseases based on the available data (G2). It was in this context that the research question was formulated, and the investigation sought to provide an answer.

The literature review examined existing PTMs in the same field, identifying seven models derived from or based on the BERT family. These models addressed the essential considerations, including dominance and performance, as observed in similar studies.

However, several gaps were identified. For instance, training models in a clinical context, particularly in the area of cardiology and applied to medical imaging reports, is a challenging endeavour. The models identified had a more general application and underperformed due to the lack of specificity of the vocabulary and the medical context. Another drawback was the absence of Portuguese-language trained models for this context, with only the Albertina family models being notable.

To address existing gaps, this research selected MediAlbertina, a model specifically trained on Portuguese clinical data, along with other models from the Albertina family and BERT-based architectures, including BERTimbau, BlueBERT, Albertina (900 and 1500), MediAlbertina (900 and 1500, and RoBERTa. Fine-tuning processes were applied to transform unstructured reports into structured data, incorporating tokenization, part-of-speech tagging and manual annotation.

In terms of performance, MediAlbertina 1500 demonstrated the highest accuracy (95.04%), followed by Albertina 900 (94.13%) and Albertina 1500 (94.02%). By contrast, RoBERTa achieved only 78.03%, emphasising the importance of domain-specific adaptations for medical datasets.

Hyperparameter tuning further improved results, with MediAlbertina 1500 achieving 96.13% accuracy and Albertina 1500 reaching 95.5%, alongside reduced validation loss. These enhancements, confirmed through confusion matrix analyses, established the MediAlbertina models as the most effective for clinical entity recognition tasks in the Portuguese language.

While the hyperparameter tuning process yielded significant improvements in model performance, a potential overfitting in later epochs was observed, suggesting that additional regularisation techniques and early stopping criteria should be incorporated in future iterations. This observation underscores the delicate balance required when fine-tuning complex language models for specialised domains such as medical text analysis.

Overall, the classification of class “O” demonstrated high accuracy, and the identification of diseases and diagnoses exhibited a solid performance, with classes “B-D”, “I-D”, “B-R” and “I-R” being well classified in the majority of cases.

Furthermore, hyperparameter tuning allowed for an evaluation of the models’ efficiency, highlighting Albertina 900 as the most efficient model.

During the deployment phase, the outcomes of this research were translated into a dynamic and interactive report developed in Power BI. By leveraging the MediAlbertina model for NER, entities were extracted and structured into a data frame, which was then exported to a CSV file to feed the report.

This integration allowed for the creation of visuals that quickly identify prevalent diseases, understand their distribution by age and type of examination and establish associations between diseases and diagnoses. As a result, this tool facilitates faster and more efficient medical interventions, saving both time and resources, and potentially improving patient outcomes.

The general limitations of the research can be grouped into two main categories. Firstly, the computer resources needed to fine-tune the PTMs require a significant investment of time and memory. The general limitations of the research can be grouped into two main categories. Firstly, the computational resources required to fine-tune the PTMs demand a significant investment of time and memory. Secondly, the annotated corpus used for clinical validation could be more comprehensive, but the availability of specialised expertise, such as medical professionals for annotation, is limited, which poses a challenge to ensuring the robustness of the solution.

The research achieved its intended objectives, proving that applying NLP techniques to medical documents can result in notable enhancements in the precision and efficacy of heart disease identification. From the implementation of language PTMs to the practical application stage, these advancements were transformed into valuable and useful tools in real-world settings.

Finally, considering all the examination and the research question, which has been based on the literature review, it was possible to identify language models trained in European Portuguese, specifically adapted to the medical lexicon, which have shown very promising results in NER, namely for entities such as diseases and diagnoses. The effectiveness of these models has been demonstrated throughout this research. For example, models such as MediAlbertina 1500, the latest iteration of the Albertina family, have shown impressive accuracy in identifying and classifying these entities in cardiology-related medical imaging reports.

The NER capabilities of these models have not only proven to be technically robust given their promising performance metrics but also have significant practical applications. Specifically, the results of the MediAlbertina 1500 hyperparameter tuning model were used to generate an interactive report in Power BI. This report is an essential tool in the patient selection process, allowing a detailed and dynamic visualisation of the extracted medical information, which in turn facilitates more efficient and faster analysis of medical data.

In conclusion, this research demonstrates that language models trained in European Portuguese and adapted to the clinical domain are highly effective in performing NER for entities such as diseases and diagnoses in medical reports. By fine-tuning these models with an annotated corpus, their high applicability and success in this specific context was showcased. Furthermore, the results suggest that fine-tuning or even hyperparameter optimisation can enhance their performance, making these models even more promising for practical applications. While this study focused on Portuguese, the methodology and findings can be extended to other languages by employing a similar approach with annotated corpora. These models have significant potential to improve the screening process and support clinical decision-making, highlighting their broader relevance in healthcare.

### Future work

Based on the results obtained and the limitations identified during the research, several promising avenues for future work have been identified that could expand the impact of the current investigation and address the gaps highlighted.

Firstly, a significant challenge was identified in the form of the need for substantial computational resources to train the models. Future research could explore more efficient training methods, such as federated learning techniques or model compression.

These approaches could reduce the computational cost without compromising accuracy, thereby improving the feasibility of using these models in clinical settings with limited resources and speeding up large-scale implementation^86,87^.

Additionally, clinical validation should be more complete in future research. While this study relied on manual validation with healthcare professionals, further studies should involve cardiology specialists to ensure greater reliability in real medical decision-making.

It is also essential to consider the variation in medical report styles and terminology across different practitioners. Future work should test the model with diverse datasets to assess its robustness and adaptability across various clinical scenarios.

Moreover, testing the model with new datasets related to cardiology and ensuring accurate annotation will be beneficial. Adjusting the model to new data structures and analysing its results can offer valuable insights into its applicability. It is also pertinent to evaluate the proposed model using medical reports from various hospitals, aiming to validate the promising results achieved. Ablation studies evaluating the impact of key preprocessing steps, such as manual annotation and tokenization, could provide deeper insights into their individual contributions to model performance. Future research could systematically analyse how different preprocessing techniques influence NER performance in clinical texts.

In conclusion, the present research has established a robust foundation upon which further investigation can be conducted in multiple directions. By addressing the limitations identified and exploring novel techniques and approaches, future research has the potential not only to enhance the accuracy and efficiency of NLP models applied to medical reports but also to expand their applicability in diverse clinical contexts. This could lead to significant advances at the intersection of technology and health, ultimately contributing to improved patient care and decision-making.

## Data Availability

The data that support the findings of this study are available from Hospital Santa Maria but restrictions apply to the availability of these data, which were used under license for the current study, and so are not publicly available. Data are however available from the corresponding authors upon reasonable request and with permission of Hospital Santa Maria.
